# Telenursing with elderly people in home care service during the COVID-19 pandemic: quasi-experimental study*

**DOI:** 10.1590/1518-8345.7138.4320

**Published:** 2024-08-30

**Authors:** Maria Auxiliadora Rodrigues, Rosimere Ferreira Santana, Ana Beatriz Serra Hercules, Patricia de Fátima Augusto Barros, Clelia Barboza Lima

**Affiliations:** 1 Universidade Federal Fluminense, Escola de Enfermagem Aurora de Afonso Costa, Niterói, RJ, Brazil.; 2 University of Central Florida, Orlando, FL, United States of America.

**Keywords:** Telenursing, COVID-19, Home Care Services, Health of the Elderly, Caregivers, Health Informatics

## Abstract

**Objective:**

to evaluate telenursing as a support technology in the transition of care for elderly people and their caregivers in the context of home care during the COVID-19 pandemic.

**Method:**

quasi-experimental before-after, non-randomized study, with 219 elderly people and caregivers from the home care service, divided into 131 in the intervention groups and 88 in the control group. Analytical treatment, descriptive and inferential statistics were carried out.

**Results:**

1691 calls were made, 1515 to the intervention group and 176 to the control group. It was observed that in the first call there is a greater need for interventions to promote health and this quantity decreases throughout the calls with a significant result (p-value < 0.001). The outcomes analyzed were hospitalization, death, discharge or continuation of the home care service and it was observed that the chance of discharge from the service was nine times greater in the intervention group. Continuity of care from the home care service and discharge after the end of the calls were also significant (p-value < 0.001).

**Conclusion:**

telenursing was a technology to support care, mainly for health promotion and discharge from home care services.

## Highlights

**(1)** Telenursing contributes to the transition process of care during a pandemic scenario and for the elderly.

**(2)** Discharge from home care service was nine times higher in the intervention group.

**(3)** Reduction in the number of interventions throughout the application of telenursing.

**(4)** Accessible and easy-to-use technology applied to both the elderly and the caregiver.

**(5)** Liaison/intervention instrument based on nursing theory and process.

## Introduction

With the worsening of the COVID-19 pandemic, due to the dissipation of the Coronavirus Disease-19 (COVID-19), which developed so quickly and dramatically, health services collapsed and were unable to respond quickly to the routine of the work process. The beginning of this health chaos began in December 2019, from bronchoalveolar lavage samples obtained from patients with pneumonia of unknown cause in Wuhan, Hubei province, China, when the betacoronavirus called Severe Acute Respiratory Syndrome Coronavirus-2 (Sars-Cov-2)^1^ ^)-(^ ^3^ was discovered.

During the COVID-19 pandemic, there was greater concern about the elderly population because they represent 31.2 million people in Brazil, corresponding to almost 15% of the total population^4^ and, due to the aging process, there is a compromised of the immune system which, when associated with chronic diseases intrinsic to age, can promote increased vulnerability to severe forms of COVID-19, leading to death^5^. This was proven with the first confirmed death being that of a 62-year-old man, diagnosed with diabetes mellitus and high blood pressure^6^ and with studies that demonstrated that the highest fatality rate in Brazil was among the age group over 60, representing 69.3% of confirmed deaths in 2020 and 64% of these deaths had at least one risk factor such as heart disease, diabetes mellitus, kidney disease, among others. The board of the World Health Organization and China presented studies that the same comorbidities in the elderly population are indicators of greater risk for mortality due to COVID-19^5^ ^),(^ ^7^.

Given this scenario and with frontline healthcare professionals significantly affected by this new disease, an opportunity has opened up to provide long-distance healthcare services due to the high degree of transmission and contamination of the Sars-Cov-2 virus, in addition to the overload of the health network and the priority of hospital care for serious cases. This strategy is a concept known as telemedicine since the 90s and is conceptualized as the use of information and communication technologies to provide health care. This concept applied to nursing is called telenursing and has wide application and effectiveness in health care, management, assistance, teaching and research services, in addition to the advantages of expanding health coverage and reducing costs. Then, with the rapid advancement of the pandemic, national bodies were compelled to transform the telemedicine and telenursing support service into the main patient care strategy^8^ ^)-(^ ^11^.

This scenario is based on and justified by the Transition Theory, which consists of “the transition from one state, condition or location to another” and which begins through uncontrollable events^12^. In this context, the COVID-19 pandemic, which forced physical and social isolation around the world, presented itself as one of these uncontrollable events, which provided elderly people with a transition in care, more specifically from in-person to remote care. This theory has a strong influence on nursing intervention, based on transitional care with a relationship between the elderly person-caregiver-nurse planned prior to life-changing situations, with strategies in the transitional care process that improve people’s quality of life, reducing the potential risk that the transition experience can provide. In this study, the phases of the transition process were divided into the entry phase, with the beginning of the pandemic and telephone contact, passage through the telephone intervention process, and exit, in this study identified as the outcome. This outcome can be positive, which would be no contamination by COVID-19 and continuity of care for chronic comorbidities, or negative, which would be contamination or discontinuity of care, causing hospitalizations or deaths^12^ ^)-(^ ^13^.

In this theory, it is also understood that the transition is essentially positive, as in the process, greater maturity and stability are obtained and, when applied to nursing care carried out over the telephone, they tend to be apprehended and reproduced, since there is no intermediary Only the caregiver can do it. Furthermore, if this transition causes anxiety, disorientation, irritability and stress, the telephone is an easy and immediate means of communication to assist in this process and achieve success and coping, as described in transition theory^12^ ^)-(^ ^13^.

To then act on the transition from face-to-face to remote care with nursing interventions specifically aimed at elderly people and caregivers, nursing diagnoses from the NANDA-I Taxonomy^14^ were used, based on diagnoses of risk of tension in the role of the caregiver, risk of infection, and risk of frail elderly syndrome. Associated with nursing diagnoses, the NIC^15^ interventions, respectively with the NANDA-I diagnoses, were based on classifications of improved coping; caregiver support; infection control and protection against infection; assistance with self-care, essential activities of daily living, bathing/hygiene, dressing/grooming, feeding and transfer; cognitive stimulation and facilitation of self-responsibility.

Thus, in the situation of the COVID-19 pandemic, with elderly people as a risk group, consequently in a vulnerable situation, with overworked caregivers, inserted in the collapsing health system, and the advancement of telenursing in the transition from in-person care to the remote, this study aimed to evaluate telenursing as a support technology in the transition of care for elderly people and their caregivers in the context of home care during the COVID-19 pandemic. The objective outcomes of the study include discharge from the Home Care Service (HCS), which means continuity of care in primary care, hospitalization or death.

## Method

### Study design

This is an excerpt from the doctoral thesis entitled “Telenursing in the Continuity of Care for the Elderly and Caregivers in the HCS During COVID-19: a quasi-experimental study” to evaluate telenursing as a support technology in the transition of care to the elderly and their caregiver in the COVID-19 pandemic. Quasi-experimental, before-and-after, non-randomized study. In order to construct the method, CONSORT was used.

### Study setting

The research was developed at HCS in São Gonçalo, RJ, Brazil.

### Period

The study was carried out from March to May 2020.

### Population

The study population was made up of elderly people registered with the HCS and their caregivers.

Due to the COVID-19 pandemic and the cooperation agreement established with the HCS, a telephone directory was provided with all registered patients and care provided by that service, which comprised 392 people, including children, adults and elderly people.

### Selection criteria

The inclusion criteria were: being registered with the HCS; being 60 years old or over. If you have a caregiver: being the main caregiver of the elderly person accompanied by the HCS; agree to participate in the research; being able to respond to the researcher’s questions in a coherent manner and having a landline or cell phone.

The discontinuation criteria applied: not answering the first call after three attempts; not answering at least 25% of telephone calls after the start of the nursing teleconsultation.

It should be noted that one of the criteria for being registered with a home care service of the Unified Health System is having a caregiver^16^, so all registered people had caregivers linked to the registration form, who could be family or professional, but in practice not all of them had their caregivers present at the time of the call or omitted the information from the service so that they could receive health care even without a caregiver, so the instrument was applied according to who answered the call.

### Sample definition and data collection

The sample size was determined by carrying out a sampling methodology that estimated the proportion of home care service outcome results in two populations, in this case: control and intervention. In this sense, whether *p*_*1*_ is the proportion of discharges from the home care service in the control group and whether *p*_*2*_ is the proportion of cases in the intervention group, it is necessary to measure the difference *p*_*1*_ - *p*_*2*_ , also known as the difference in risks^17^. For the calculation, information from pilot studies and previous information from already known or published studies were necessary^18^. In the pilot study, we found the following values *p*_*1*_ =0.05 and *p*_*2*_ =0.20, approximately. It was then assumed that in the intervention group the discharge rate would be four times that of the control group. Considering these values and a variation in the test’s confidence level of 95%, it was obtained that the minimum sample would be 188 elderly people, making it clear that the population of the present study (219 participants) is satisfactory.

The telephone directory provided had the name and age of the 392 people registered in the HCS in alphabetical order. These were divided in the form of a random draw into two different envelopes, named control group and intervention group, which contained 196 names in each. The envelopes were opened and, after applying the inclusion criteria, 302 elderly people eligible for the study were identified, 90 of whom were excluded, 73 from the control group and 17 from the intervention group. After the first call, 10 elderly people from the control group and 4 from the intervention group did not answer the first call after three attempts on different days and times, considering these as losses of participants, resulting in 288 included in the study, 113 in the control group and 175 in the intervention group.

**Figure 1:**
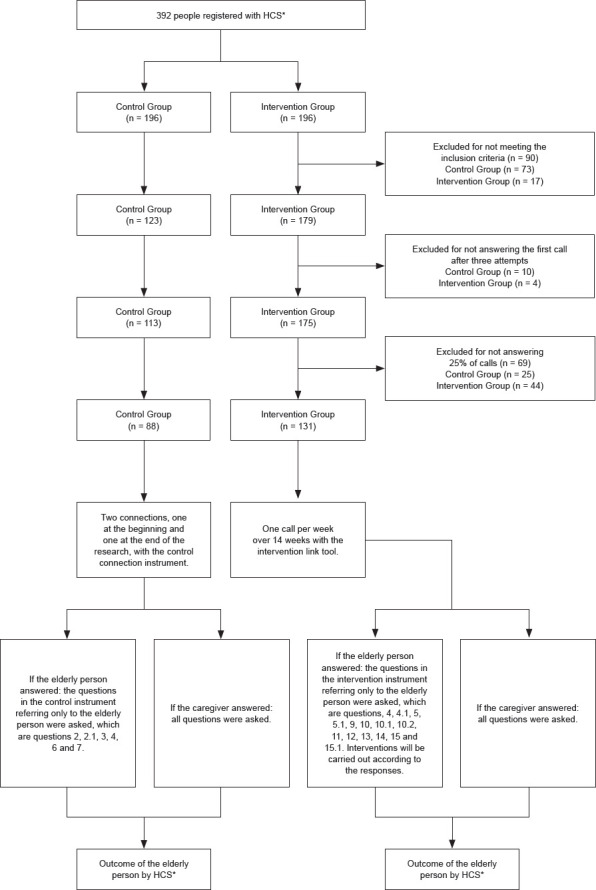
Flowchart of participants and research intervention protocol applied to the control and intervention groups *HCS = Home Care Service

During the study, 69 elderly people were excluded for not answering 25% of telephone calls, 44 in the intervention group and 25 in the control group, resulting in a final sample of 219 elderly people, 131 in the intervention group, 88 in the control group (Figure 1).

### Instruments

The research team, formed by the main researcher, in addition to two nurses and two nursing students, created two connection instruments to be used in telephone contact, one for the intervention group and one for the control group (Figure 2), which served to guide the telephone calls made.

**Figure 2:** Control and intervention group research instrument for elderly people and caregivers

Control Instrument		
NUMBER	QUESTION	LEGEND
CARE		Self-care
		Non-Formal
		Formal
CAREGIVER		Self-care
		Spouse
		Son
		Father/mother
		Grandson
		Brother
		Nephew
		Cousin
		Daughter-in-law
		Neighbor
		Formal Caregiver
CONDITION OF THE ELDERLY PERSON - How are you? What about the elderly person? How many people are in the house? How many people on the ground?		Descriptive
HOUSE RESIDENTS - How many residents are there in the house?		Number of residents
QUESTION 1	How is the care of elderly people at home in times of social distancing to prevent coronavirus?	Calm
		Can carry out all planned activities on the day
		Tiring
		Running
		Unable to carry out planned activities on the day
		Mental stress
		No food or supplies
		No medicine
QUESTION 2	Was there a change in the routine to adapt to the care of elderly people?/Was there an increase in care demands during the pandemic?	No
		Yes
QUESTION 3	Is the elderly person isolated?	No
		Yes
QUESTION 4	Does anyone in the house work? And do they leave the house to go to work?	Nobody works
		Works in Essential Activities and leaves the house
		Works in Non-Essential Activities and leaves the house
		Works in Non-Essential Activities and works from home
QUESTION 5	How is the health of the elderly person?	No symptoms/all well
		Shortness of breathe
		Cough
		Tiredness
		Pain
		Fever
		Runny nose
		No sense of smell and/or taste
QUESTION 5.1	For how long?	Number in days
QUESTION 6	Did the elderly person take a rapid COVID test?	No
		Yes
QUESTION 6.1	If so, what is the result?	Negative
		Positive
QUESTION 6.2	If positive, what is the result?	Not applicable
		IGM* positive
		IGG^†^ positive
QUESTION 7	Is the caregiver isolated?	No
		Yes
		Not applicable
QUESTION 8	How is the caregiver’s health?	No symptoms/all well
		Shortness of breathe
		Cough
		Tiredness
		Pain
		Fever
		Runny nose
		No sense of smell and/or taste
		Not applicable
QUESTION 8.1	For how long?	Number in days
QUESTION 9	Did the caregiver take a rapid COVID test?	No
		Yes
		Not applicable
QUESTION 9.1	If so, what is the result?	Did not take the test/does not apply
		Negative
		Positive
QUESTION 9.2	If positive, what is the result?	Not applicable
		IGM* positive
		IGG^†^ positive
QUESTION 10	How is the health of the other people in the house?	No symptoms/all well
		Shortness of breathe
		Cough
		Tiredness
		Pain
		Fever
		Runny nose
		No sense of smell and/or taste
		Not applicable
QUESTION 10.1	For how long?	Number in days
QUESTION 11	Has anyone else in the house taken the rapid test?	No
		Yes
QUESTION 11.1	If yes, who? Consider people other than elderly people and caregivers. Example: if the caregiver was the spouse, this question should not be considered, but if the caregiver was the child and the husband took the test, this question should be answered.	Not applicable
		Elderly person’s spouse
		Elderly person’s son
		Elderly grandson
		Elderly person’s parents
		Brother of the elderly
		Caregiver’s spouse
QUESTION 11.2	If so, what is the result?	Didn’t take the test
		Negative
		Positive
QUESTION 11.3	If positive, what is the result?	Not applicable
		IGM* positive
		IGG^†^ positive
QUESTION 12	Has the elderly person already been vaccinated against the flu?	No
		Yes
QUESTION 12.1	Who vaccinated?	HCS^‡^
		FHU^§^
QUESTION 13	Has the caregiver already been vaccinated against the flu?	0 Not applicable
		1 No
		2 Yes
QUESTION 13.1	Who vaccinated?	Not applicable
		HCS^‡^
		FHU^§^
QUESTION 14	Have you received visits from HCS? How often?	No
		Weekly
		Every 15 days
		Monthly
QUESTION 15	Was there use of health services?	No
		Yes
OUTCOME		Continued HCS^‡^ care after calls ended
		He no longer wanted to receive the HCS^‡^ face-to-face visit because of the pandemic, nor the research calls (he signed the form and did not withdraw from the research)
		HCS^‡^ discharge after calls or termination of calls
		Hospitalization
		Death
Reason for hospitalization or death		Descriptive
**Intervention Instrument**		
**COLUMN**	**QUESTION**	**LEGEND**
CAREGIVER		Self-care
		Non-Formal
		Formal
TYPE OF CAREGIVER		Self-care
		Spouse
		Son
		Father/mother
		Grandson
		Brother
		Nephew
		Cousin
		Daughter-in-law
		Neighbor
		Formal Caregiver
CONDITION OF THE ELDERLY PERSON - How are you? What about the elderly person? How many people are in the house? How many people on the ground?		Descriptive
CAREGIVER STATUS		Descriptive
RESIDENTS OF THE HOUSE - How many residents are there in the house?		Number of people living in the house
QUESTION 1	How is the care of elderly people at home in times of social distancing to prevent coronavirus?	Calm
		Can carry out all planned activities on the day
		Tiring
		Running
		Unable to carry out planned activities on the day
		Mental stress
		No food or supplies
		No medicine
INTERVENTION 1		Use a calm, reassuring approach.
INTERVENTION 2		Encourage the verbalization of feelings, perceptions and fears
INTERVENTION 3		Provide the caregiver with realistic information about aspects of caring for the elderly person.
INTERVENTION 4		Evaluate the possibility of rotating care
INTERVENTION 5		Encourage caregiver dialogue with family members regarding care
QUESTION 1.1	If the answer is Tiring, Rushing or Unable to carry out the activities planned for the day: Why does the Lord say this	Descriptive
QUESTION 2	How do you feel about the care provided to elderly people during this pandemic?	Calm/tranquil
		Satisfied
		Sad
		Tired out
		Overloaded
		Sold off
		Distressed
		Stressed
		Insecurity
QUESTION 2.1	How often?	No, never
		Rarely
		Sometimes
		Often
		Ever
INTERVENTION 6		Encourage family participation online as appropriate
INTERVENTION 7		Request services from other healthcare professionals for the patient as appropriate
INTERVENTION 8		Act on behalf of the caregiver when overload becomes evident/Explain the operating system of the health care network, such as the call to the core of the multidisciplinary support team that will offer support to the caregiver and the family in social and psychological matters.
INTERVENTION 9		Encourage leisure activities at home (You can send material via WebWhats Comercial)
QUESTION 3	Is the elderly person isolated?	No
		Yes
QUESTION 4	How is the health of the elderly person?	No symptoms/all well
		Shortness of breathe
		Cough
		Tiredness
		Pain
		Fever
		Runny nose
		No sense of smell and/or taste
QUESTION 4.1	How much time?	Number in days
QUESTION 5	Did the elderly person take a rapid COVID test?	No
		Yes
QUESTION 5.1	If so, what is the result?	Negative
		Positive
QUESTION 5.2	If positive, what is the result?	Not applicable
		IGM* positive
		IGG^†^ positive
QUESTION 6	Is the caregiver isolated?	No
		Yes
		Not applicable
QUESTION 7	How is the caregiver’s health?	No symptoms/all well
		Shortness of breathe
		Cough
		Tiredness
		Pain
		Fever
		Runny nose
		No sense of smell and/or taste
		Not applicable
QUESTION 7.1	How much time?	Number in days
QUESTION 8	Did the caregiver take a rapid COVID test?	No
		Yes
		Not applicable
QUESTION 8.1	If so, what is the result?	Negative
		Positive
		Not applicable
QUESTION 8.2	If positive, what is the result?	Not applicable
		IGM* positive
		IGG^†^ positive
QUESTION 9	How is the health of family members?	No symptoms/all well
		Shortness of breathe
		Cough
		Tiredness
		Pain
		Fever
		Runny nose
		No sense of smell and/or taste
		Not applicable
QUESTION 9.1	How much time?	Number in days
QUESTION 10	Has anyone else in the house taken the rapid test?	No
		Yes
QUESTION 10.1	If yes, who? *Consider people other than the elderly person and caregiver. Example: if the caregiver was the spouse, this question should not be considered, but if the caregiver was the child and the husband took the test, this question should be answered.*	Not applicable
		Elderly person’s spouse
		Elderly person’s son
		Elderly grandson
		Elderly person’s parents
		Brother of the elderly
		Caregiver’s spouse
QUESTION 10.2	If so, what is the result?	Didn’t take the test
		Negative
		Positive
QUESTION 10.3	If positive, what is the result?	Not applicable
		IGM* positive
		IGG^†^ positive
INTERVENTION 10		When you have mild symptoms, advise care at home - SEE COVID-19 care flow
QUESTION 11	Who does the shopping?	They don’t leave the house/order by phone/order via app
		The elderly person themselves
		The caregiver
		Elderly person’s spouse
		Elderly person’s son
		Elderly grandson
		Elderly person’s parents
		Brother of the elderly
		Elderly nephew
		Elderly person’s son-in-law
		Elderly daughter-in-law
		Elderly person’s neighbor
QUESTION 12	Does anyone in the house work? And do you leave the house to work?	Nobody works
		Work in Essential Activities and leave the house
		Work in Non-Essential Activities and leave the house
		Works in Non-Essential Activities and works from home
QUESTION 13	Has anyone travelled in the last 14 days?	No
		Yes
QUESTION 14	Contacts in the last 14 days?	No
		Yes
QUESTION 15	Has the elderly person already been vaccinated against the flu?	No
		Yes
QUESTION 15.1	Who vaccinated them?	Not applicable
		HCS^‡^
		FHU^§^
QUESTION 16	Has the caregiver already been vaccinated against the flu?	No
		Yes
		Not applicable
QUESTION 16.1	Who vaccinated them?	Not applicable/Not taken
		HCS^‡^
		FHU^§^
INTERVENTION 11		Explain the difference between flu and corona and the protection that vaccines offer.
QUESTION 17	Was there a change in the routine to adapt to the care of the elderly person/their care?	No
		Yes
INTERVENTION 12		Teach the caregiver strategies for maintaining health care in order to reduce contamination (hygiene and antisepsis)
INTERVENTION 13		Teach how to maintain your own physical and mental health (sleep, exercises at home, relaxation music, nutrition)
INTERVENTION 14		Determine the need for improvements at home to compensate for hygiene and mental health, detail
INTERVENTION 15		Determine needs for changes related to home security detail
INTERVENTION 16		Provide guidance on suitable conditions for living with elderly people, detail
INTERVENTION 17		When coughing and sneezing, cover your mouth and nose with a disposable tissue and throw it in the trash, or protect it with your forearm. Do not use your hands in order to avoid transmitting the virus to objects and people;
INTERVENTION 18		Wash your hands with soap and water for at least 20 seconds frequently. When this is not possible, clean and sanitize your hands with 70% alcohol gel;
INTERVENTION 19		Avoid close contact with people who have cold or flu symptoms;
INTERVENTION 20		Avoid close contact with elderly people, pregnant women, women who are breastfeeding or people who get sick easily;
INTERVENTION 21		Do not share personal objects, such as glasses, cutlery and plates, and clean objects that are frequently touched, such as cell phones.
QUESTION 18	Do you have any of the care you provide for elderly people who are having difficulty?	None
		To have a bath
		Personal hygiene: using the bathroom, cleaning yourself or tidying your clothes
		Dress up
		Feeding
		Transfer: Get up and walk
		Band Aid
INTERVENTION 22		Person to undertake self-care activities
INTERVENTION 23		Guide the caregiver to encourage the elderly person to take maximum responsibility for their own self-care
INTERVENTION 24		Support the caregiver in setting limits and caring for themselves
INTERVENTION 25		Teach the caregiver strategies for maintaining health care in order to maintain their own physical and mental health
INTERVENTION 26		Encourage the verbalization of feelings, perceptions and fears regarding bathing
INTERVENTION 27		Guide the caregiver to establish a trusting relationship with the elderly person when bathing
INTERVENTION 28		Guide the caregiver to encourage the elderly person to verbalize signs and symptoms related to pain, sensitivity, itching, changes in hearing, tinnitus, vertigo, changes in muscle strength, skin integrity and edema.
INTERVENTION 29		Instruct the caregiver to wash and condition the elderly person’s hair, massaging the scalp with their fingertips, and giving preference to using liquid and neutral soap when bathing.
INTERVENTION 30		Instruct the caregiver to clean the nails, apply creams to the skin, especially bony prominences, comb the hair with wide combs, carefully dry the body folds and between the fingers, and keep the perineum dry and clean at regular intervals.
INTERVENTION 31		Guide the caregiver regarding the signs and symptoms of urinary retention/urinary infection, which are decreased frequency, consistency, odor, volume, color, pain and bladder globe as appropriate.
INTERVENTION 32		Guide care regarding the occurrence of signs and symptoms of diarrhea, constipation, impaction and fecal incontinence.
INTERVENTION 33		Instruct the caregiver that with each vesico-intestinal complication it is necessary to perform adequate hygiene (soap and water).
INTERVENTION 34		Instruct the caregiver to provide the elderly person with the necessary clothing in an accessible area.
INTERVENTION 35		person to undertake self-care activities
INTERVENTION 36		Instruct the caregiver to assist the elderly person with regard to the use of drawstrings, buttons and zippers.
INTERVENTION 37		Instruct the caregiver to encourage the elderly person to dress themselves according to their physical condition.
INTERVENTION 38		Guide the caregiver to encourage the elderly person to verbalize about their limitations in dressing
INTERVENTION 39		Instruct the caregiver to monitor the condition of the elderly person’s mouth: lips, tongue, mucous membranes, teeth, gums and dental appliances and their adaptation. Pay attention to signs of inflammation, spongy, retracted or bleeding gums; dry, cracked lips; wounds; raw, scarlet tongue; and hyperemia and hypertrophy of the papillae.
INTERVENTION 40		Instruct the caregiver to correctly position the elderly person to facilitate chewing and swallowing, providing appropriate support for the neck.
INTERVENTION 41		Instruct the caregiver to offer the food in an appetizing way, at the appropriate temperature, arranged on the plate, if necessary, cutting the meat, peeling the eggs, when opening packages and/or packaged foods.
INTERVENTION 42		Instruct the caregiver to offer cutlery and devices according to the elderly person’s needs, such as straws and spoons.
INTERVENTION 43		Guide the caregiver to help and/or encourage the elderly person to promote their oral hygiene routine.
INTERVENTION 44		Advise the caregiver to observe non-verbal cues of discomfort, such as facial expressions and excessive movement in elderly people who are unable to communicate effectively.
INTERVENTION 45		Guide the caregiver or family to obtain tools to assist with daily activities: walker, cane, crutches, support bars, adequate lighting, round corners and railings if necessary.
INTERVENTION 46		Guide the caregiver or family to control environmental factors that may negatively influence mobility, such as room temperature, heat or cold, lighting and noise.
INTERVENTION 47		Guide the caregiver to reduce factors that precipitate or increase the experience of pain, such as fear, fatigue, monotony and lack of information.
INTERVENTION 48		Advise the appropriate use of prescribed analgesics to help with pain when it is related to movement.
INTERVENTION 49		Advise changing position every 2 hours, protecting bony prominences and avoiding edema.
INTERVENTION 50		Instruct the caregiver to encourage the elderly person to get involved in position changes when possible.
INTERVENTION 51		Instruct the caregiver to keep the elderly person’s bed clean and with sheets stretched to avoid tension in wounds and shear at the time of mobilization.
INTERVENTION 52		Instruct the caregiver to use appropriate equipment to support limbs and bony prominences, such as cushions.
QUESTION 19	Did the elderly person develop any different behavior during this social distancing?	None/no
		Unrest
		Sadness
		Aggressiveness
		Confusion
		Memory disorders
		Orientation disorders
		Behavior disorders
		Anxiety
		Fear
INTERVENTION 53		Orientation for the patient concerning time, space and person.
INTERVENTION 54		Provide a calendar.
INTERVENTION 55		Stimulate cognition through exercises such as painting, drawing, puzzles, singing and listening to music, using television and radio, posting photos of the patient’s close family members.
INTERVENTION 56		Provide guidance on the environment in which the elderly person sleeps, darken the environment when it is time to sleep, have adequate ventilation.
INTERVENTION 57		Use calming techniques such as music the elderly person prefers, touch them and be present.
INTERVENTION 58		Recognize the patient’s fears and feelings.
INTERVENTION 59		Allow the elderly person to maintain some of their rituals to limit anxiety
INTERVENTION 60		Inform the caregiver’s perception in a calm, reassuring and non-argumentative way.
INTERVENTION 61		Responding to the tone, rather than the content, of the hallucination or delusion.
INTERVENTION 62		Maintain a well-lit environment that reduces sharp contrasts and shadows.
QUESTION 20	Was there use of health services?	No
		Yes
OUTCOME		Continued HCS^‡^ care after calls ended
		He no longer wanted to receive a face-to-face visit from HCS^‡^ because of the pandemic nor the research calls (he signed the form and did not withdraw from the research)
		HCS^‡^ discharge after calls or termination of calls
		Hospitalization
		Death
Reason for hospitalization or death		Descriptive

*IGM = Immunoglobulin M; ^†^IGG = Immunoglobulin G; ^‡^HCS = Home Care Service; ^§^FHU = Family Health Unit

The instrument in Figure 1 was developed in light of gerontological assessment and based on Transition Theory^12^ ^),(^ ^19^. It was drawn up in a systematic way with the aim of developing an adequate plan of care and support in the intervention of the general condition of the elderly person and their caregiver related to the care of both during the investigation of the flu syndrome and social isolation in times of COVID-19.

The telecare questions and interventions were prepared in accordance with the legal duties of the nursing professional, based on the diagnoses of risk of tension in the caregiver role, risk of infection and risk of frail elderly syndrome from the NANDA-I Taxonomy^14^, associated with these diagnoses, respectively, the NIC^15^ interventions, based on the classifications of improved coping; caregiver support; infection control and protection against infection; assistance with self-care, essential activities of daily living, bathing/hygiene, dressing/grooming, feeding and transfer; cognitive stimulation and facilitation of self-responsibility and in the Coronavirus (COVID-19) Clinical Management Protocol in Primary Health Care of the Ministry of Health^20^. Regarding the comparison of assessments and interventions in the health of elderly people and their caregivers through teleconsultation, during the COVID-19 pandemic, the NANDA I and NIC domains were used.

These diagnoses and interventions were chosen in view of the COVID-19 pandemic to promote and prevent the health of elderly people and their care in social isolation and aiming to control COVID-19 infection. 

When a situation was identified at the time of the telephone call that required urgency/emergency or in-person assistance, the HCS team was contacted by the research team and provided appropriate assistance or referral.

This instrument was validated by five gerontology experts in March 2020 and it has been applied to seven elderly people registered in the home care service who were not included in the study before the start of data collection and subsequently preliminary data were published indicating the validation of the instrument^21^.

### Data processing and analysis

The data were tabulated using Microsoft Excel 2016 software. They were then imported into the statistical software IBM-SPSS (Statistical Package for the Social Sciences) version 24, for due analytical treatment. Descriptive statistics were used, arranged in table form, where the frequencies and percentages of the variables under study were recorded. After initial data assessments, statistical methods were applied to verify associations and correlations between variables. To evaluate the behavior between two categorical variables, Fisher’s statistical test (Association Test) was used, and, when evaluating differences between means, the Kruskal-Wallis test was applied. 

### Ethical aspects

The research was approved by the Human Research Ethics Committee of the research host institution with the Certificate of Presentation of Ethical Appreciation (CAAE) number 14354919.1.0000.5243 and 43682821.6.0000.5243. Technical cooperation agreement signed with the municipality to which the HCS was linked.

Regarding the recruitment of participants, the research team made the first telephone contact to explain the research that would be carried out, initially obtaining verbal consent to participate by reading the Free and Informed Consent Form (FICF) for both the elderly person and the caregiver. At the time, the confidentiality of the information was highlighted. Subsequently, during the in-person visit by the HCS team in partnership with the research team, the signature of the Free and Informed Consent Form was obtained from all participants.

There were no direct risks to the participants since the nursing interventions, offered through telephone calls, were already part of the scope of guidelines and nursing care already offered to the HCS population. A slight discomfort was observed, which may have been caused by inconvenience related to the calls and possible emotional changes regarding the questions and interventions in the questionnaire, which were minimized by the researchers with active listening and offering psychological or medical support from the multidisciplinary team of the HCS. The researchers’ telephone contact was made available in case of complications, guidance and referrals. Nursing care related to health promotion was offered through guidelines regarding hygiene, crowding, respect for social isolation and maintenance of better living conditions during the COVID-19 pandemic with improved emotional and functional capacity.

The benefits of the study deal with the social, academic and professional contribution to the consolidation of innovative nursing care in the public body, low cost and easy to manage, used to support the service of nursing teams, promoting assistance in health promotion, adherence treatment, psychological comfort, screening and prevention of health complications in elderly people.

After the end of the study, the control group received all calls with the appropriate nursing interventions in the same period.

## Results

The present study included a total of 1691 calls, of which 1515 calls were made to the 131 participants in the intervention group and 176 calls to the 88 participants in the control group, using the appropriate call instruments for each group.

The results in Table 1 detail the characteristics of the study participants, as well as the separation by group analyzed (control and intervention). Fisher’s Exact test was applied to evaluate the percentage distribution of the characteristic in relation to the analyzed groups, that is, its homogeneity.

**Table 1:** Assessment of the homogeneity of the sample of elderly people and caregivers. São Gonçalo, RJ, Brazil, 2020

Characteristic	Grupo				Total		P-value*
	Control		Intervention				
	n	%	n	%	N	%	
**Elderly gender**							0,165
Male	44	50,0	52	39,7	96	43,8	
Female	44	50,0	79	60,3	123	56,2	>0,999
**Caregiver gender**							
Male	13	15,7	17	15,2	30	15,4	
Female	70	84,3	95	84,8	165	84,6	
**Age (average)**							
Elderly person	75,2		75,9				0,849
Caregiver	51,6		53,1				0,615
**Has a caregiver**							
Yes	83	94,3	112	85,5	195	89,0	0,048
No	5	5,7	19	14,5	24	11,0	0,068
**Number of comorbidities in elderly people**							
0	0	0,0	4	3,1	4	1,8	
1	7	8,0	14	10,7	21	9,6	
2	21	23,9	37	28,2	58	26,5	
3	22	25,0	29	22,1	51	23,3	
4	13	14,8	28	21,4	41	18,7	
5	11	12,5	14	10,7	25	11,4	
6	6	6,8	4	3,1	10	4,6	
7	4	4,5	1	0,8	5	2,3	
8	3	3,4	0	0,0	3	1,4	
9	1	1,1	0	0,0	1	0,5	0,709
**Number of comorbidities in caregivers**							
0	44	50,0	70	53,4	114	52,1	
1	28	31,8	34	26,0	62	28,3	
2	15	17,0	23	17,6	38	17,4	
3	1	1,1	4	3,1	5	2,3	

*Fisher’s exact test, significant if p-value < 0.05

Among the characteristics analyzed for homogeneity, the only one that showed significance was “Has a caregiver”, since percentage differences were observed between the groups (p-value = 0.048), indicating that the control group had a higher percentage of elderly people with a caregiver, 94.3%, compared to the intervention, 85.5%.

An assessment of the homogeneity of quantitative variables was carried out regarding: age, caregiver’s age, number of elderly people’s comorbidities and number of caregivers’ comorbidities, through statistical summaries and application of the Kruskal-Wallis test, with the aim of evaluating whether the distribution of values between the control and intervention groups were similar. It is noteworthy that the Kruskal-Wallis test is significant if the p-value is < 0.05.

In this sense, it was observed that only the comparison ‘Number of comorbidities in elderly people’ in the control group was greater than in the intervention group (p-value = 0.023). The remaining comparisons were not significant.

In relation to health assessments of elderly people and caregivers regarding the signs and symptoms of COVID-19, namely: shortness of breath, cough, tiredness, pain, fever, runny nose and absence of taste and/or smell, during the calls, the Kruskal-Wallis test was also applied with significance if the p-value was < 0.05. In this evaluation, there were no percentage differences between the control and intervention groups.

Regarding the comparison of the caregiver’s health assessments in relation to the groups analyzed, significance was observed in the “No symptoms” option, favoring the control group (p - value < 0.001). This had more positive evaluations than the intervention group.

When comparing residents’ health assessments in relation to the groups analyzed, the most positive result was “no symptoms” in the intervention group (p-value < 0.001).

Table 2 shows the average number of interventions carried out with the elderly person and caregiver during calls in the intervention group.

**Table 2:** Average number of interventions carried out in the intervention group with elderly people and caregivers. São Gonçalo, RJ, Brazil, 2020

Call week	Domain 7 - Roles and Relationship				Domain 11 - Safety/security				Domain 1 - Health promotion			
	Diagnosis - Risk of Caregiver Role Strain				Diagnosis - Risk of Contamination				Diagnosis - Risk of Frail Elderly Syndrome			
	Median	95% CI*		P-value^§^	Median	95%CI*		P-value^§^	Median	95% CI*		P-value^§^
		Bottom^†^	Superior^‡^			Bottom^†^	Superior^‡^			Bottom^†^	Superior^‡^	
1	4,73	4,39	5,06	**<0,001**	8,97	8,67	9,27	**<0,001**	4,99	4,02	5,96	**<0,001**
2	2,07	1,82	2,32		3,19	2,67	3,72		0,26	0,1	0,42	
3	1,85	1,62	2,08		2,41	1,93	2,88		0,15	0,04	0,27	
4	1,68	1,45	1,91		2,02	1,62	2,42		0,14	0,02	0,26	
5	1,6	1,4	1,81		1,5	1,25	1,76		0,1	0	0,2	
6	1,5	1,32	1,68		1,57	1,25	1,89		0,13	0	0,25	
7	1,47	1,3	1,65		1,62	1,28	1,95		0,04	0	0,11	
8	1,45	1,29	1,61		1,58	1,23	1,94		0,07	0	0,16	
9	1,45	1,29	1,62		1,57	1,21	1,93		0,02	0	0,06	
10	1,48	1,3	1,66		1,73	1,34	2,13		0,01	0	0,03	
11	1,49	1,31	1,67		1,71	1,29	2,13		0,03	0	0,09	
12	1,56	1,34	1,78		1,83	1,38	2,27		0,03	0	0,09	
13	1,69	1,38	1,99		1,82	1,38	2,26		0,02	0	0,06	
14	1,63	1,36	1,89		1,81	1,34	2,27		0,02	0	0,07	

*CI = Confidence interval; ^†^Bottom = Statistics; ^‡^Superior = Statistics; ^§^P-value = Fisher’s Exact Test, significant if p-value < 0.05

It is observed that at the first connection there is a greater need for interventions. Over the course of the calls, this quantity decreases and this result was significant (p-value < 0.001) for all the items studied, but mainly for health promotion, which deals with fragility and these stabilize over time, while Safety/ Protection remained something necessary and was reinforced with the COVID-19 pandemic, associated with the promotion of caregiver well-being included in the Roles and Relationships domain.

In Table 3, the existence of a difference in the percentage of responses for each outcome is assessed in relation to the control and intervention groups. There is a difference in the outcomes analyzed (p-value < 0.001). It is observed that in the discharge outcome, the result was percentage significant in the intervention group, so of the cases that were discharged from HCS^(36)^, almost 84% were in the intervention group. It is important to highlight that the discharge from HCS was carried out by the service team as the research progressed and the patient’s evaluations were positive.

**Table 3:** Comparison between outcome of the control group and intervention for elderly people and caregivers. São Gonçalo, RJ, Brazil, 2020

Outcome	Group				Total		P-value*
	Control		Intervention				
	n	%	n	%	n	%	
1 Continued with HCS^†^ care after the calls ended	67	44,4	84	55,6	151	100	**<0,001**
2 HCS^†^ discharge after calls or end of calls	6	16,7	30	83,3	36	100	
3 Hospitalization	11	64,7	6	35,3	17	100	
4 Death	4	26,7	11	73,3	15	100	

*P-value = Fisher’s Exact Test, significant if p-value < 0.05; ^†^HCS = Home Care Service

To consider these results in the best way, the use of the Odds Ratio was applied and these results were compared with the “discharge” outcome with the other outcomes. In this analysis, it was observed that the chance of discharge in the intervention group was higher when compared to other outcomes in the control group. Thus, having a “high” result is almost nine times more likely to occur in the intervention group than in the control group.

## Discussion

Given the moment of transition in health experienced in the world due to the COVID-19 pandemic, consecutively in the lives of the binomial elderly people and their caregivers served by HCS, it provided the possibility of using technological resources mediated by telenursing, in supporting the continuity of care for elderly people and their caregivers assisted by the service.

In an unprecedented way, the study was developed at the height of the COVID-19 pandemic, based on the scientificity of the combination of the Transition Theory^12^ ^),(^ ^19^, the NANDA I Standardized Language System^14^ and NIC^15^ for documental guidance of the connection instrument and also the regulation of COFEN Resolution 696/2022^22^. The combination of transition theory through response patterns, indicators of the process of caring for elderly people and their caregivers, feeling connected to professionals, interacting even over the telephone, being situated and developing trust and coping enabled the foundation of data collection.

The standardized languages of the NANDA I^14^ and NIC^15^ Taxonomies were crucial in identifying the main Nursing diagnoses such as: Fragile Elderly Syndrome, Risk of Caregiver Role Strain and Risk of Contamination and the NIC Taxonomy , the main interventions based on the respective diagnoses.

International studies in the 1990s already showed that approximately half of all patients who go to home care need continuity of qualified nursing care. These highlight that, at the time of discharge from hospital, patients are still very weak and have not recovered enough to learn the necessary self-care procedures^23^.

With this, the home care environment emerges through TeleHomeCare Technology; that is, the studies also predicted that this technology would help provide the necessary level of care and services for home care to older people and their caregivers by registered home care nurses proficient in TeleHomeCare. In view of this, studies have already highlighted that in situations of public calamity, creativity and the search for remote care strategies overcome the challenges imposed by distancing measures and become part of the agenda of health systems in different care contexts^23^.

Due to the state of public calamity and the study proposal, it was possible to unite the research team and the HCS multidisciplinary team through training, associate with the use of technology and put into practice the nursing care planning contained in the five uninterrupted stages of the Nursing Process systematized and composed in the connection instrument. Telenursing made it possible to provide innovative support and continuity assistance during the COVID-19 Pandemic, for elderly people and their caregivers who were hospitalized at home and at this time of social isolation, did not have access to specific health services. 

It is worth mentioning that health professionals needed to be qualified to develop innovative skills/competencies to guarantee the quality, safety and efficiency of care through technology. This justifies the creation of the call instrument based on the transition from in-person to remote care during the COVID-19 pandemic^24^.

As with other regulations on access to society’s data, telenursing must also be previously consented to by the patient or their legal representative, and its implementation is the responsibility of the nurse. Registration of the teleconsultation was mandatory and based on the nursing process (nursing consultation, diagnosis, planning, intervention and evaluation), to organize and guide care for elderly people and their caregivers. Thus, in the calls, elderly people and their caregivers at HCS were approached regarding data collection based on the combination of transition theory, diagnoses were extracted from the NANDA-I^14^ domains: family roles and relationships, safety/ health protection and promotion.

In domain I Health Promotion, the diagnosis Risk for Frail Elderly Syndrome was extracted, because, despite there being no consensual definition, frailty can be understood as a multidimensional syndrome, which involves the complex interaction of biological, psychological and social factors. This interaction culminates in greater vulnerability and is associated with the risk of clinical outcomes such as functional decline, falls, malnutrition, hospitalization and death. Therefore, a nursing care plan regarding these signs and symptoms was essential. The NIC interventions were related to the nursing diagnosis regarding the prevention of acute complications, guiding the adoption of a balanced diet, personal care and staying active^25^.

In Domain 7 Roles and Relationships, the justification was also given through better assistance coverage for elderly people and their caregivers in the COVID-19 pandemic. In this domain, it was due to the possible physical and emotional overload of the elderly person’s caregiver. NIC interventions were justified with the objective of minimizing the deleterious effects of tension in the caregiver’s role through the nursing guidelines provided in the connection instrument. In domain 11 Security and Protection, the Risk of Infection during the COVID-19 pandemic stood out. Interventions were also guided in a systematic way through the connection instrument.

During the calls, it was identified and confirmed that the elderly people and their caregivers had chronic illnesses and were monitored by the HCS and the municipality’s Health Care Network without any regularity. Studies corroborate that with the worsening of the COVID-19 pandemic, the care effort in general increased significantly and this was reflected in the physical and mental health of the population, especially women who, almost one in two, have chronic comorbidities that worsened during the pandemic. And, consecutively, the feelings of isolation, sadness and anxiety on the part of this binomial, elderly people and their caregivers, were also increased^26^ ^)-(^ ^27^. 

This data is in line with the present study, considering the high percentage of elderly people who had more female caregivers than male caregivers, as shown in Table 1. There was also identification of the female gender for caregivers who did not receive remuneration, which is in line with of studies on the care provided to elderly people^28^.

In the calls made to elderly people and their caregivers, it was possible to observe the need for a different perspective from the State, society and families regarding organization and family planning. Studies already narrate the importance of including the caregiver not only as an executor of planned actions, but as the main subject in the promotion and quality of care, when the elderly person loses the condition of self-care.

The visibility of the caregiver’s role was relevant in the study since, through it, it was possible to provide health care to Binomial, an elderly person and caregiver. Public health policies are necessary to cover comprehensive care for the health of elderly people and their caregivers, after all, the caregiver is on the “anonymous front line” in caring for elderly people, not only during the COVID-19 pandemic, but as a requirement for the elderly person’s adherence to the Home Care Service^29^.

Telenursing brought about a significant number of interventions in the first call and was maintained during the first four weeks due to the panic that set in among citizens, due to the uncertainties and deaths that were being caused by the pandemic. The pandemic state did not make it possible for the HCS team to visit in person and health care for the population in general was hampered.

In this sense, telenursing as support in nursing interventions in the continuity of care for elderly people and their caregivers, was innovative and essential to resolve patients’ doubts, alleviate the fear of interaction which, according to the news published in the media, were all predictor of deaths, in this episode it was possible for the liaison team to develop their nursing actions masterfully and acquire the trust of the elderly person and their caregivers assisted by HCS.

After four weeks, it can be observed that the number of calls decreased in terms of diagnosing the Risk of Fragile Elderly Syndrome and was equivalent to other interventions for Risk of Contamination and Risk of Caregiver Role Strain, a positive result (p -value < 0.001).

Safety recommendations for elderly people went far beyond those already carried out daily by caregivers. It was necessary to intensify care and attention to preventive actions directly linked to behavior and hygiene, as the time was full of loneliness and doubts regarding the COVID-19 disease^19^ ^),(^ ^22^ ^),(^ ^30^ ^)-(^ ^31^. 

Other results are similar to national and international studies, with regard to elderly people and their caregivers remaining in the HCS, being discharged, being hospitalized or dying after the calls or ending the calls. There was a highlight of 83.3% for the intervention group, as at the height of COVID-19, hospitalizations were intended for more serious cases such as Acute Respiratory Failure Syndrome, which culminated in an increase in the discharge of patients from the entire Hospital Network. Health Care and also in those adhered to by HCS^26^ ^)-(^ ^27^. It is important to highlight that the discharge of the elderly person and their caregivers assisted by HCS during the research was possible through the updated information that the research team transmitted to the HCS multidisciplinary team and this also provided continuity of care through technology until possible high.

Given the significant results, the importance of health professionals in minimizing the effects caused by the public health calamity of the COVID-19 pandemic was clear, especially nursing workers, protagonists in controlling the transmission of this disease, both on the front line and in interventions to support the continuity of care for elderly people and their caregivers through telenursing.

Therefore, the results showed that telenursing carried out by nurses as a support technology in the transition of care to elderly people and their caregivers during the COVID-19 pandemic obtained a confidence index of 95% and significance (p-value < 0.001) in all cases. domains and diagnoses studied (Roles and Relationships - Risk of Caregiver Role Strain; Safety and Protection - Risk of Contamination and Health Promotion - Risk of Frail Elderly Syndrome). The importance of telenursing in interventions carried out by the nursing team stands out, with positive results in preventing the health of elderly people and their caregivers.

In terms of information, safeguarding the information and records obtained during telenursing is the responsibility of the professional who performed it and/or the linked health institution. The practice of telenursing requires adjustments from the parties involved - nurse and patient - that go beyond infrastructure and connectivity^21^. In this sense, the nurse forwards information to the HCS team to certify the death.

“Doing health” is carried out by the nursing team or multidisciplinary team in the most diverse contexts, so that each professional category has its own framework of knowledge and practices that aim to diagnose, prevent and recover from diseases and injuries that affect the individual. , the family and the community. It is important to know that telenursing must be developed by a nursing professional with technical competence and autonomy and who knows how to distinguish an advanced nursing practice from a generalist one^32^. 

The support of transitions theory was essential to support therapeutic objectives by pointing out the results of indicators of the care process: feeling connected, interacting, being situated and developing trust and coping. Mastery was considered, because, according to the theory, in the occurrence of fluid integration between the team of nurses in supporting the care of the elderly person and caregivers, essential, in the face of crisis situations such as the pandemic, care for the elderly person became doubled as it is the group most susceptible to complications from COVID-19. This was confirmed by the reduction in the number of interventions after the first few weeks.

Regarding the limitations of the study, it can be said that at the height of the pandemic, access to data on elderly people and their caregivers registered in the HCS were out of date, which made it impossible to randomize the study. These limitations led the research team to assist the HCS team in updating the records of elderly people and their caregivers affiliated with the HCS, concomitantly with the research. The scarcity of data regarding COVID-19 was also a limitation.

Regarding the implications for the advancement of scientific knowledge in the area of health and nursing, it was observed that telenursing can be responsible for positive and negative implications when developed by lay people as a call center. Nursing care through telenursing can be carried out throughout the public and private health care network for elderly people, their caregivers and other citizens who require nursing interventions. Technology to support nurses demands technical and scientific skills necessary for assertive clinical decision-making. Linkage instruments based on scientific evidence are useful for directing care planning for elderly people and their caregivers during the COVID-19 pandemic. The connections must be clearly based on the disease and the role of the nurse in terms of longitudinal care for the elderly person and their caregivers assisted by the Home Care System or other health niches.

Telenursing, despite already existing in the world, only during the COVID-19 pandemic, was regulated in Brazil by the Cofen/Corens System on an emergency basis to support nurses in making clinical decisions during the COVID-19 pandemic. In view of this, it is essential that new research be developed into the effectiveness of telenursing as support for the continuity of integral health care for the elderly and their caregivers. The HCS, as it is a multidisciplinary assistance equipment, for the entire healthcare team, would enable monitoring the health of its registered participants with several possibilities for positive interventions to support the continuity of longitudinal care^(33)-(34)^. 

## Conclusion

The results demonstrated the relevance of the use of scientificity regarding the use of the combination of transition theory with the diagnostic taxonomy and interventions for the continuity of care planning for elderly people and their caregivers assisted by HCS, through telenursing, with the chance of increase nine times higher when receiving care over the telephone. The nursing diagnoses highlighted in this article as Risk of Caregiver Role Strain; Risk of Frail Elderly Syndrome and Risk of Contamination and the interventions contained in the connection instrument were efficient and essential, as they allowed the objective of the study to be met in the development of telenursing to support the continuity of face-to-face care for the elderly person and their relatives. caregivers focused on preventing and promoting their health in the COVID-19 pandemic. Thus, this support through telenursing also allowed the HCS multidisciplinary team to carry out a precise analysis regarding the safe discharge of patients enrolled in the service, directing them to the Primary Health Care network for low complexity care.
