# Supplementary material for: Telenursing with elderly people in home care service during the COVID-19 pandemic: quasi-experimental study
Source: Rev Lat Am Enfermagem. 2024 Aug 30;32:e4320. doi: 10.1590/1518-8345.7138.4320 (PMC11368068; doi:10.1590/1518-8345.7138.4320)
Supplement: Translation

# Translation: Telenfermagem com pessoas idosas no serviço de atenção domiciliar na pandemia de COVID-19: estudo quase-experimental*

**DOI:** 10.1590/1518-8345.7138.4321

**Authors:** Maria Auxiliadora Rodrigues, Rosimere Ferreira Santana, Ana Beatriz Serra Hercules, Patricia de Fátima Augusto Barros, Clelia Barboza Lima

**Affiliations:** 1 Universidade Federal Fluminense, Escola de Enfermagem Aurora de Afonso Costa, Niterói, RJ, Brasil.; 2 University of Central Florida, Orlando, FL, Estados Unidos da América.

**Keywords:** Telenfermagem, COVID-19, Serviços de Assistência Domiciliar, Saúde do Idoso, Cuidadores, Tecnologia em Saúde

## Abstract

**Objetivo:**

avaliar a telenfermagem como tecnologia de apoio na transição do cuidado às pessoas idosas e seus cuidadores no contexto da atenção domiciliar na pandemia COVID-19.

**Método:**

estudo quase experimental tipo antes-depois, não randomizado, com 219 pessoas idosas e com cuidadores do serviço de atenção domiciliar, divididos em 131 no grupo intervenção e 88 no grupo controle. Foi realizado tratamento analítico, estatísticas descritivas e inferenciais.

**Resultados:**

foram realizadas 1691 ligações, sendo 1515 para o grupo intervenção e 176 para o controle. Observou-se que na primeira ligação há maior necessidade de intervenções para promoção da saúde e que essa quantidade diminui ao longo das ligações com resultado significativo (p-valor < 0,001). Os desfechos analisados foram internação, óbito, alta ou continuação do serviço de atenção domiciliar e observou-se que a chance de alta do serviço foi nove vezes maior no grupo intervenção. A continuidade do cuidado do serviço de atenção domiciliar e alta após o término das ligações também foram significativos (p-valor < 0,001).

**Conclusão:**

a telenfermagem foi uma tecnologia de apoio ao cuidado, principalmente para a promoção da saúde e na alta do serviço de atendimento domiciliar.

## Destaques

**(1)** Telenfermagem contribui no processo de transição do cuidado em pandemias e ao idoso.

**(2)** Alta do serviço de atendimento domiciliar nove vezes maior no grupo intervenção.

**(3)** Diminuição do número de intervenções ao longo da aplicação da telenfermagem.

**(4)** Tecnologia acessível e de fácil utilização aplicado tanto para o idoso e ao cuidador.

**(5)** Instrumento de ligação/intervenção fundamentado em teoria e processo de enfermagem.

## Introdução

Com o agravamento da pandemia, devido à dissipação da doença *Coronavirus Disease-19* (COVID-19), que se desenvolveu de forma tão veloz e dramática, os serviços de saúde entraram em colapso e não conseguiram responder de forma rápida à rotina do processo de trabalho. O início desse caos na saúde deu-se em dezembro de 2019, a partir de amostras de lavado broncoalveolar obtidas de pacientes com pneumonia de causa desconhecida em Wuhan, província de Hubei, China, quando foi descoberto o betacoronavírus denominado *Severe Acute Respiratory Syndrome Coronavirus-2* (Sars-Cov-2)^1^ ^)-(^ ^3^. 

Nesse período, houve uma maior preocupação com a população idosa, que representa 31,2 milhões de pessoas no Brasil, correspondendo a quase 15% da população total^4^, e que, devido ao processo de envelhecimento, possui um comprometimento do sistema imunológico, principalmente quando associado às doenças crônicas também relativas à idade, podendo promover um aumento da vulnerabilidade às formas graves da COVID-19, inclusive, podendo evoluir para o óbito^5^. Isso se comprovou quando o primeiro óbito confirmado foi o de um homem de 62 anos, diagnosticado com diabetes mellitus e hipertensão arterial^6^ ^)^ e, também, a partir de estudos que demonstraram que a maior letalidade no Brasil estava entre a faixa etária de mais de 60 anos, representando 69,3% dos óbitos confirmados em 2020. Desses óbitos, 64% apresentavam pelo menos um fator de risco como: cardiopatia, diabetes mellitus, doença renal, entre outras. A junta da Organização Mundial da Saúde e a China apresentaram estudos sobre as mesmas comorbidades na população idosa, demonstrando que são indicadores de maior risco para mortalidade com a COVID-19^5^ ^),(^ ^7^.

Com os profissionais de saúde da linha de frente afetados de forma significativa por essa nova doença, abriu-se uma oportunidade para realizar atendimentos de saúde a longa distância, considerando o distanciamento como medida de proteção devido ao alto grau de transmissão e contaminação do vírus Sars-Cov-2, além da sobrecarga da rede de saúde e da prioridade de atendimento hospitalar aos casos graves. Tal estratégia é conhecida como telemedicina, desde os anos 90, e caracteriza-se pelo uso das tecnologias da informação e comunicação para a prestação de cuidados em saúde. O conceito, aplicado à enfermagem, é chamado de telenfermagem e tem ampla aplicação e eficácia nos serviços de atenção à saúde, gestão, assistência, ensino e pesquisa, além das vantagens de ampliar a cobertura de saúde e de reduzir custos. Assim, com o avanço rápido da pandemia, os órgãos nacionais foram impelidos a transformar o serviço de telemedicina e telenfermagem - de um serviço de apoio para a principal estratégia de atendimento ao paciente^8^ ^)-(^ ^11^.

Tal cenário fundamenta-se e justifica-se na Teoria da Transição, que consiste na “passagem de um estado, condição ou local, para outro”. Assim, ela se inicia através de eventos incontroláveis^12^. Nesse contexto, a pandemia de COVID-19 forçou o isolamento físico e social no mundo, apresentando-se como um desses eventos incontroláveis, de forma a proporcionar às pessoas idosas uma transição no cuidado, mais especificamente do cuidado presencial para o remoto. A teoria exerce forte influência na intervenção de enfermagem, a partir de um cuidado transicional com um relacionamento entre pessoa idosa-cuidador-enfermeiro planejado anteriormente a situações de mudanças na vida, com estratégias no processo do cuidado de transição que melhore a qualidade de vida das pessoas, diminuindo o risco potencial que a experiência de transição pode proporcionar. As fases do processo de transição dividiram-se em: fase de entrada, com o início da pandemia e do contato telefônico; passagem pelo processo de intervenção telefônica; e saída, identificado como desfecho neste estudo. O desfecho pode ser positivo - que seria a não contaminação por COVID-19 e a continuidade do cuidado de comorbidades crônicas - ou negativo - que seria a contaminação ou descontinuidade do cuidado, provocando internações ou óbitos^12^ ^)-(^ ^13^.

Entende-se que a transição é essencialmente positiva, pois no processo dela obtém-se maior maturidade e estabilidade e quando aplicado aos cuidados de enfermagem realizados por telefone, tendem a ser apreendidos e reproduzidos, já que não há um intermediário para realizá-lo, somente o cuidador. Além disso, caso essa transição cause ansiedade, desorientação, irritabilidade e estresse, o telefone é uma via fácil e imediata de comunicação para auxiliar no processo e obter-se sucesso e *coping*, conforme descreve a teoria da transição^12^ ^)-(^ ^13^.

Sendo assim, para atuar na transição do cuidado presencial para o remoto com as intervenções de enfermagem orientadas especificamente para as pessoas idosas e cuidadores, utilizou-se os diagnósticos de enfermagem de Taxonomia da NANDA-I^14^, baseados nos diagnósticos de risco de tensão do papel do cuidador, risco de infecção, risco de síndrome do idoso frágil. Associados aos diagnósticos de enfermagem, as intervenções do NIC^15^, respectivamente aos diagnósticos NANDA-I partiram das classificações de melhora do enfrentamento; apoio ao cuidador; controle de infecção e proteção contra infecção; assistência do autocuidado, das atividades essenciais da vida diária, banho/higiene, vestir/arrumar-se, alimentação e transferência; estimulação cognitiva e facilitação da autorresponsabilidade.

Dessa forma, considerando o dado contexto pandêmico, com as pessoas idosas como grupo de risco, consequentemente estando em situação de vulnerabilidade, com cuidadores sobrecarregados, inseridos no sistema de saúde em colapso, assim como levando em conta o avanço da telenfermagem na transição do cuidado presencial para o remoto, este estudo teve por objetivo avaliar a telenfermagem como tecnologia de apoio na transição do cuidado às pessoas idosas e seus cuidadores, no contexto da atenção domiciliar, na pandemia COVID-19. Os desfechos do objetivo do estudo incluem alta do serviço de atenção domiciliar (SAD), que significa continuidade do cuidado na atenção básica, internação ou óbito.

## Método

### Tipo do estudo

Trata-se de um recorte da tese de doutorado intitulada “Telenfermagem na Continuidade do Cuidado ao Idoso e Cuidador no SAD Durante a COVID-19: estudo quase experimental” para avaliar a telenfermagem como tecnologia de apoio na transição do cuidado à pessoa idosa e seu cuidador na pandemia de COVID-19. Estudo quase experimental do tipo antes e depois, não randomizado. Para construção do método, foi utilizado o CONSORT.

### Local do estudo

A pesquisa foi desenvolvida no SAD em São Gonçalo, RJ, Brasil.

### Período

O estudo foi realizado no período de março a maio de 2020.

### População

A população do estudo foi composta por pessoas idosas cadastrados no SAD e seus cuidadores.

Dado o acordo de cooperação estabelecido com o SAD, foi fornecida uma lista telefônica com todos os pacientes cadastrados e com os atendimentos realizados por aquele serviço, os quais compreendiam 392 pessoas, incluindo crianças, adultos e pessoas idosas. 

### Critérios de seleção

Os critérios de inclusão foram: estar cadastrados ao SAD; ter idade igual ou superior a 60 anos. Se possuir cuidador: ser o principal cuidador da pessoa idosa acompanhada pelo SAD; aceitar participar da pesquisa; ser capaz de responder aos questionamentos realizados pela pesquisadora de forma coerente e possuir telefone fixo ou celular. 

Os critérios de descontinuidade aplicados: não atender à primeira ligação após três tentativas; não atender a, no mínimo, 25% das chamadas telefônicas após o início da teleconsulta de enfermagem.

Ressalta-se que um dos critérios para estar cadastrado a um serviço de atendimento domiciliar do Sistema Único de Saúde é ter um cuidador^16^, então todas as pessoas cadastradas possuíam cuidadores vinculados na ficha cadastral, podendo ser familiar ou profissional, mas na prática nem todos estavam com os cuidadores presentes no momento da ligação ou omitiram a informação ao serviço para que pudessem receber o cuidado em saúde mesmo sem ter o cuidador, sendo assim o instrumento foi aplicado de acordo com quem atendia a ligação.

### Definição da amostra e coleta de dados

O tamanho da amostra foi determinado com a execução de metodologia amostral que estimou a proporção de resultados dos desfechos do serviço de atenção domiciliar em duas populações, no caso: controle e intervenção. Nesse sentido seja *p*_*1*_ a proporção de altas do serviço de atenção domiciliar no grupo controle e seja *p*_*2*_ a proporção de casos no grupo intervenção, é necessário medir a diferença *p*_*1*_ -*p*_*2*_ , também conhecida como diferença dos riscos^17^. Para o cálculo, foram necessárias informações de estudos-piloto e de informações prévias de estudos já conhecidos ou publicados^18^. No estudo-piloto, encontramos os seguintes valores *p*_*1*_ =0,05 e *p*_*2*_ =0,20, aproximadamente. Supôs-se então que, no grupo intervenção, a proporção de alta seria o quádruplo do grupo controle. Considerando esses valores e uma variação do nível de confiança do teste de 95%, obteve-se que a amostra mínima seria de 188 pessoas idosas, restando evidente que a população do presente estudo (219 participantes) é satisfatória.

A lista telefônica fornecida possuía nome em ordem alfabética e idade das 392 pessoas cadastradas no SAD. Estas foram divididas em forma de sorteio aleatório em dois envelopes distintos, nomeados grupo controle e grupo intervenção, que contiveram 196 nomes em cada. Abriram-se os envelopes e, após aplicação dos critérios de inclusão, foram identificadas 302 pessoas idosas elegíveis para o estudo, sendo 90 excluídas, 73 do grupo controle e 17 do grupo intervenção. Após primeira ligação, 10 pessoas idosas do grupo controle e 4 do grupo intervenção não atenderam a primeira ligação após três tentativas em dias e horários diferentes, considerando essas como perdas de participantes, resultando em 288 incluídos no estudo, sendo 113 no grupo controle e 175 no grupo intervenção. 

No decorrer do estudo, 69 pessoas idosas foram excluídas por não atenderem 25% das ligações telefônicas, sendo 44 do grupo intervenção e 25 do controle, obtendo-se uma amostra final de 219 pessoas idosas, sendo 131 no grupo intervenção, 88 no controle (Figura 1).

**Figura 1:** Fluxograma de participantes e protocolo de intervenção da pesquisa aplicado aos grupos controle e intervenção *SAD = Serviço de Atenção Domiciliar

### Instrumentos

A equipe de pesquisa, formada pela pesquisadora principal, além de duas enfermeiras e duas alunas de enfermagem, criou dois instrumentos de ligação a serem utilizados no contato telefônico, sendo um para o grupo intervenção, e um para o grupo controle (Figura 2), os quais serviram para guiar as ligações telefônicas realizadas.

**Figura 2:** Instrumento de pesquisa do grupo controle e intervenção para pessoa idosa e cuidadores

Instrumento de Controle		
NÚMERO	PERGUNTA	LEGENDA
CUIDADOR		Autocuidado/o próprio
		Não Formal
		Formal
TIPO DE CUIDADOR		Autocuidado/o próprio
		Cônjuge
		Filho(a)
		Pai/mãe
		Neto(a)
		Irmão(ã)
		Sobrinho(a)
		Primo(a)
		Nora
		Vizinha
		Cuidador Formal
ESTADO DA PESSOA IDOSA - Como o senhor (a) está? E a pessoa idosa? Quantas pessoas estão na casa? Quantas pessoas no terreno?		Descritiva
MORADORES DA CASA - Quantos moradores tem na casa?		Número de moradores
PERGUNTA 1	Como está o cuidado da pessoa idosa em casa, em tempos de distanciamento social pela prevenção do coronavírus?	Tranquilo
		Consegue realizar todas as atividades planejadas no dia
		Cansativo
		Corrido
		Não consegue realizar as atividades planejadas no dia
		Estresse mental
		Sem comida ou mantimentos
		Sem remédio
PERGUNTA 2	Houve mudança na rotina para se adequar ao cuidado da pessoa idosa? Houve aumento nas demandas do cuidado durante a pandemia?	Não
		Sim
PERGUNTA 3	A pessoa idosa está isolada?	Não
		Sim
PERGUNTA 4	Alguém na casa trabalha? E sai de casa para trabalhar?	Ninguém trabalha
		Trabalha em Atividades Essenciais e sai de casa
		Trabalha em Atividades Não Essenciais e sai de casa
		Trabalha em Atividades Não Essenciais e está de home office
PERGUNTA 5	Como está a saúde da pessoa idosa?	Nenhum sintoma/todos bem
		Falta de ar
		Tosse
		Cansaço
		Dores
		Febre
		Coriza
		Sem olfato e/ou paladar
PERGUNTA 5.1	Quanto tempo?	Número em dias
PERGUNTA 6	A pessoa idosa fez o teste rápido do COVID?	Não
		Sim
PERGUNTA 6.1	Se sim, qual o resultado?	Negativo
		Positivo
PERGUNTA 6.2	Se positivo, qual o resultado?	Não se aplica
		IGM^*^ positivo
		IGG^†^ positivo
PERGUNTA 7	Cuidador está isolado?	Não
		Sim
		Não se aplica
PERGUNTA 8	Como está a saúde do cuidador?	Nenhum sintoma/todos bem
		Falta de ar
		Tosse
		Cansaço
		Dores
		Febre
		Coriza
		Sem olfato e/ou paladar
		Não se aplica
PERGUNTA 8.1	Quanto tempo?	Número em dias
PERGUNTA 9	O cuidador fez o teste rápido de COVID?	Não
		Sim
		Não se aplica
PERGUNTA 9.1	Se sim, qual o resultado?	Não fez o teste/não se aplica
		Negativo
		Positivo
PERGUNTA 9.2	Se positivo, qual o resultado?	Não se aplica
		IGM* positivo
		IGG^†^ positivo
PERGUNTA 10	Como está a saúde das outras pessoas da casa?	Nenhum sintoma/todos bem
		Falta de ar
		Tosse
		Cansaço
		Dores
		Febre
		Coriza
		Sem olfato e/ou paladar
		Não se aplica
PERGUNTA 10.1	Quanto tempo?	Número em dias
PERGUNTA 11	Mais alguém da casa fez o teste rápido?	Não
		Sim
PERGUNTA 11.1	Se sim, quem? *Considerar pessoas além de pessoa idosa e cuidador. Exemplo: se o cuidador foi o cônjuge essa pergunta não deve ser considerada, mas se o cuidador for o filho e o marido tiver feito o teste, essa pergunta deve ser respondida.*	Não se aplica
		Cônjuge da pessoa idosa
		Filho da pessoa idosa
		Neto da pessoa idosa
		Pais da pessoa idosa
		Irmão da pessoa idosa
		Cônjuge do cuidador
PERGUNTA 11.2	Se sim, qual o resultado?	Não fez o teste
		Negativo
		Positivo
PERGUNTA 11.3	Se positivo, qual o resultado?	Não se aplica
		IGM* positivo
		IGG^†^ positivo
PERGUNTA 12	A pessoa idosa já foi vacinada contra gripe?	Não
		Sim
PERGUNTA 12.1	Quem vacinou?	SAD^‡^
		USF^§^
PERGUNTA 13	Cuidador já foi vacinado contra gripe?	0 Não se aplica
		1 Não
		2 Sim
PERGUNTA 13.1	Quem vacinou?	Não se aplica
		SAD^‡^
		USF^§^
PERGUNTA 14	Tem recebido visita do SAD? Com que frequência?	Não
		Semanalmente
		A cada 15 dias
		Mensal
PERGUNTA 15	Houve uso de serviços de saúde?	Não
		Sim
DESFECHO		Continuou com o cuidado do SAD^‡^ após o término das ligações
		Não quis mais receber a visita presencial do SAD^‡^ por causa da pandemia e nem as ligações da pesquisa (assinou o termo e não retirou a participação da pesquisa)
		Alta do SAD^‡^ após as ligações ou término das ligações
		Internação
		Óbito
Motivo da internação ou óbito		Descritivo
**Instrumento de Intervenção**		
**COLUNA**	**PERGUNTA**	**LEGENDA**
CUIDADOR		Autocuidado/o próprio
		Não Formal
		Formal
TIPO DE CUIDADOR		Autocuidado/o próprio
		Cônjuge
		Filho(a)
		Pai/mãe
		Neto(a)
		Irmão(ã)
		Sobrinho(a)
		Primo(a)
		Nora
		Vizinha
		Cuidador Formal
ESTADO DA PESSOA IDOSA - Como o senhor (a) está? E a pessoa idosa? Quantas pessoas estão na casa? Quantas pessoas no terreno?		Descritivo
ESTADO DO CUIDADOR		Descritivo
MORADORES DA CASA - Quantos moradores têm na casa?		Número de moradores
PERGUNTA 1	Como está o cuidado da pessoa idosa em casa em tempos de distanciamento social pela prevenção do coronavírus?	Tranquilo
		Consegue realizar todas as atividades planejadas no dia
		Cansativo
		Corrido
		Não consegue realizar as atividades planejadas no dia
		Estresse mental
		Sem comida ou mantimentos
		Sem remédio
INTERVENÇÃO 1		Usar uma abordagem calma, tranquilizadora.
INTERVENÇÃO 2		Encorajar a verbalização de sentimentos, percepções e medos
INTERVENÇÃO 3		Fornecer ao cuidador informações realistas sobre os aspectos dos cuidados a pessoa idosa.
INTERVENÇÃO 4		Avaliar possibilidade de revezamento dos cuidados
INTERVENÇÃO 5		Incentivar diálogo do cuidador com familiares a respeito dos cuidados
PERGUNTA 1.1	Se a resposta for *Cansativo, Corrido ou Não consegue realizar as atividades planejadas no dia:* Por que o Senhor diz isso	Descritivo
PERGUNTA 2	Qual o seu sentimento relacionado ao cuidado prestado a pessoa idosa nesse tempo de pandemia?	Calmo/tranquilo
		Satisfeito
		Triste
		Cansado
		Sobrecarregado
		Esgotado
		Angustiado
		Estressado
		Insegurança
PERGUNTA 2.1	Com qual frequência?	Não/Nunca
		Raramente
		Às vezes
		Quase Sempre
		Sempre
INTERVENÇÃO 6		Encorajar a participação da família *online*, conforme apropriado
INTERVENÇÃO 7		Solicitar serviços de outros profissionais de saúde para o paciente, conforme apropriado
INTERVENÇÃO 8		Agir em prol do cuidador quando a sobrecarga se tornar evidente/Explicar sobre o sistema de funcionamento da rede de assistência a saúde, como a chamada no núcleo da equipe multiprofissional de apoio que oferecerão suporte ao cuidador e a família na questão social e psicológica.
INTERVENÇÃO 9		Incentivar a realização de atividades de lazer no domicílio (Pode enviar material pelo *WebWhats* Comercial).
PERGUNTA 3	A pessoa idosa está isolada?	Não
		Sim
PERGUNTA 4	Como está a saúde da pessoa idosa?	Nenhum sintoma/todos bem
		Falta de ar
		Tosse
		Cansaço
		Dores
		Febre
		Coriza
		Sem olfato e/ou paladar
PERGUNTA 4.1	Quanto tempo?	Número em dias
PERGUNTA 5	A pessoa idosa fez o teste rápido do COVID?	Não
		Sim
PERGUNTA 5.1	Se sim, qual o resultado?	Negativo
		Positivo
PERGUNTA 5.2	Se positivo, qual o resultado	Não se aplica
		IGM* positivo
		IGG^†^ positivo
PERGUNTA 6	Cuidador está isolado?	Não
		Sim
		Não se aplica
PERGUNTA 7	Como está a saúde do cuidador?	Nenhum sintoma/todos bem
		Falta de ar
		Tosse
		Cansaço
		Dores
		Febre
		Coriza
		Sem olfato e/ou paladar
		Não se aplica
PERGUNTA 7.1	Quanto tempo?	Número em dias
PERGUNTA 8	O cuidador fez o teste rápido do COVID?	Não
		Sim
		Não se aplica
PERGUNTA 8.1	Se sim, qual o resultado?	Negativo
		Positivo
		Não se aplica
PERGUNTA 8.2	Se positivo, qual o resultado?	Não se aplica
		IGM* positivo
		IGG^†^ positivo
PERGUNTA 9	Como está a saúde dos membros da família?	Nenhum sintoma/todos bem
		Falta de ar
		Tosse
		Cansaço
		Dores
		Febre
		Coriza
		Sem olfato e/ou paladar
		Não se aplica
PERGUNTA 9.1	Quanto tempo?	Número em dias
PERGUNTA 10	Mais alguém da casa fez o teste rápido?	Não
		Sim
PERGUNTA 10.1	Se sim, quem? *Considerar pessoas além da pessoa idosa e cuidador. Exemplo: se o cuidador foi o cônjuge essa pergunta não deve ser considerada, mas se o cuidador for o filho e o marido tiver feito o teste, essa pergunta deve ser respondida.*	Não se aplica
		Cônjuge da pessoa idosa
		Filho da pessoa idosa
		Neto da pessoa idosa
		Pais da pessoa idosa
		Irmão da pessoa idosa
		Cônjuge do cuidador
PERGUNTA 10.2	Se sim, qual o resultado?	Não fez o teste
		Negativo
		Positivo
PERGUNTA 10.3	Se positivo, qual o resultado?	Não se aplica
		IGM* positivo
		IGG^†^ positivo
INTERVENÇÃO 10		Quando tiver sintomas leves orientar os cuidados em casa - VER fluxo de atendimento COVID-19
PERGUNTA 11	Quem faz as compras?	Não saem de casa/pedem por telefone/pedem por aplicativo
		A própria pessoa idosa
		O cuidador
		Cônjuge da pessoa idosa
		Filho da pessoa idosa
		Neto da pessoa idosa
		Pais da pessoa idosa
		Irmão da pessoa idosa
		Sobrinho da pessoa idosa
		Genro da pessoa idosa
		Nora da pessoa idosa
		Vizinho da pessoa idosa
PERGUNTA 12	Alguém na casa trabalha? E sai de casa para trabalhar?	Ninguém trabalha
		Trabalha em Atividades Essenciais e sai de casa
		Trabalha em Atividades Não Essenciais e sai de casa
		Trabalha em Atividades Não Essenciais e está de *home office*
PERGUNTA 13	Alguém viajou últimos 14 dias?	Não
		Sim
PERGUNTA 14	Contactantes nos últimos 14 dias?	Não
		Sim
PERGUNTA 15	A pessoa idosa já foi vacinada contra gripe?	Não
		Sim
PERGUNTA 15.1	Quem vacinou?	Não se aplica
		SAD^‡^
		USF^§^
PERGUNTA 16	Cuidador já foi vacinado contra gripe?	Não
		Sim
		Não se aplica
PERGUNTA 16.1	Quem vacinou?	Não se aplica/Não tomou
		SAD^‡^
		USF^§^
INTERVENÇÃO 11		Explicar sobre diferença entre gripe e corona e a proteção que as vacinas oferecem.
PERGUNTA 17	Houve mudança na rotina para se adequar ao cuidado da pessoa idosa/seu cuidado?	Não
		Sim
INTERVENÇÃO 12		Ensinar ao cuidador estratégias de manutenção dos cuidados de saúde de modo a diminuir a contaminação (higiene e antissepsia).
INTERVENÇÃO 13		Ensinar a manter a própria saúde física e mental (sono, exercícios em casa, música relaxamento, alimentação).
INTERVENÇÃO 14		Determinar as necessidades de melhorias em casa para compensação da higiene e da saúde mental, detalhar.
INTERVENÇÃO 15		Determinar as necessidades de mudanças relacionadas à segurança em casa, detalhar.
INTERVENÇÃO 16		Orientar sobre condições adequadas para moradia com a pessoa idosa, detalhar.
INTERVENÇÃO 17		Quando tossir e espirrar, cubra a boca e o nariz com um lenço de papel descartável e jogue-o no lixo, ou proteja com o antebraço. Não use suas mãos, a fim de evitar transmitir o vírus para objetos e pessoas.
INTERVENÇÃO 18		Lave as mãos com água e sabão por pelo menos 20 segundos, com frequência. Quando não for possível, limpe e higienize suas mãos com álcool gel 70%.
INTERVENÇÃO 19		Evite contato próximo com pessoas que estejam com sintomas de resfriado ou gripe.
INTERVENÇÃO 20		Evite contato próximo com pessoas idosas, grávidas, mulheres que estejam amamentando ou pessoas que adoeçam com facilidade.
INTERVENÇÃO 21		Não compartilhe objetos de uso pessoal, como copos, talheres e pratos, e limpe objetos que sejam tocados com frequência, como celulares.
PERGUNTA 18	Dos cuidados que realizam com a pessoa idosa tem algum que está com dificuldade?	Nenhum
		Tomar banho
		Higiene pessoal: usar o banheiro, limpar-se ou arrumar as roupas
		Vestir-se
		Alimentar-se
		Transferência: Levantar-se e andar
		Curativo
INTERVENÇÃO 22		Encorajar a independência, mas auxiliar a pessoa idosa quando esta não conseguir.
INTERVENÇÃO 23		Determinar a capacidade mental e cognitiva da pessoa idosa para assumir as atividades de autocuidado.
INTERVENÇÃO 24		Orientar o cuidador a encorajar a pessoa idosa a assumir o máximo de responsabilidade pelo próprio autocuidado.
INTERVENÇÃO 25		Apoiar o cuidador no estabelecimento de limites e nos cuidados consigo mesmo.
INTERVENÇÃO 26		Ensinar ao cuidador estratégias de manutenção dos cuidados de saúde de modo a manter a própria saúde física e mental.
INTERVENÇÃO 27		Encorajar a verbalização de sentimentos, percepções e medos em relação ao banho.
INTERVENÇÃO 28		Orientar o cuidador a estabelecer uma relação de confiança com a pessoa idosa no ato do banho.
INTERVENÇÃO 29		Orientar o cuidador a encorajar a pessoa idosa a verbalizar sinais e sintomas relacionados a dor, sensibilidade, prurido, alteração na audição, zumbido, vertigem, alteração na força muscular, integridade da pele e edemas.
INTERVENÇÃO 30		Orientar o cuidador a lavar e condicionar o cabelo da pessoa idosa, massageando com a ponta dos dedos o couro cabeludo e dar preferência à utilização de sabonete líquido e neutro no banho.
INTERVENÇÃO 31		Orientar o cuidador a realizar limpeza das unhas, aplicar cremes na pele, principalmente proeminências ósseas, pentear o cabelo com pentes amplos, secar cuidadosamente as dobras corporais e entre os dedos, e manter períneo seco e limpo em intervalos regulares.
INTERVENÇÃO 32		Orientar o cuidador quanto aos sinais e sintomas de retenção urinária/infecção urinária, que são diminuição da frequência, consistência, odor, volume, cor, dor e globo vesical conforme apropriado.
INTERVENÇÃO 33		Orientar o cuidador quanto à ocorrência de sinais e sintomas de diarreia, constipação, impactação e incontinência fecal.
INTERVENÇÃO 34		Orientar o cuidador que a cada intercorrência vesico-intestinal é necessário realizar higiene adequada (água e sabão).
INTERVENÇÃO 35		Orientar o cuidador a disponibilizar as roupas necessárias a pessoa idosa em área acessível.
INTERVENÇÃO 36		Orientar o cuidador a auxiliar a pessoa idosa no que diz respeito ao uso de cordões, botões e zíperes.
INTERVENÇÃO 37		Orientar o cuidador a estimular a pessoa idosa a vestir-se sozinho de acordo com a sua condição física.
INTERVENÇÃO 38		Orientar o cuidador a estimular a verbalização da pessoa idosa sobre as suas limitações para se vestir.
INTERVENÇÃO 39		Orientar o cuidador a monitorar a condição da boca da pessoa idosa: lábios, língua, membranas mucosas, dentes, gengivas e aparelhos dentários e sua adaptação. Atenção a sinais de inflamação, gengivas esponjosas, retraídas ou com sangramento; lábios secos, rachados; feridas; língua crua, escarlate; e hiperemia e hipertrofia das papilas.
INTERVENÇÃO 40		Orientar o cuidador a posicionar corretamente a pessoa idosa a fim de facilitar a mastigação e a deglutição, fornecendo apoio apropriado para o pescoço.
INTERVENÇÃO 41		Orientar o cuidador a oferecer a comida de forma apetitosa, na temperatura adequada, arranjados no prato, se necessário, cortando a carne, descascando os ovos, na abertura dos pacotes e/ou alimentos embalados.
INTERVENÇÃO 42		Orientar o cuidador a oferecer talheres e dispositivos conforme a necessidade da pessoa idosa, como canudos e colher.
INTERVENÇÃO 43		Orientar o cuidador a auxiliar e/ou incentivar a pessoa idosa a promover a rotina de higiene bucal.
INTERVENÇÃO 44		Orientar o cuidador a observar pistas não verbais de desconforto, como expressões faciais e movimentação excessiva em pessoas idosas que são incapazes de comunicar-se efetivamente.
INTERVENÇÃO 45		Orientar o cuidador ou a família a obter ferramentas para auxiliar nas atividades do dia a dia: andador, bengala, muletas, barras de apoio, iluminação adequada, quinas boleadas e grades caso necessite.
INTERVENÇÃO 46		Orientar o cuidador ou família a controlar fatores ambientais que possam influenciar de forma negativa na mobilidade como temperatura do ambiente, calor ou frio, iluminação e ruídos.
INTERVENÇÃO 47		Orientar o cuidador a reduzir fatores que precipitem ou aumente a experiência da dor como medo, fadiga, monotonia e falta de informação.
INTERVENÇÃO 48		Orientar a utilização adequada de analgésicos prescritos para auxílio da dor quando estas estão relacionadas a movimentação.
INTERVENÇÃO 49		Orientar mudança de posição a cada 2h, protegendo proeminências ósseas e evitando edema.
INTERVENÇÃO 50		Orientar o cuidador a encorajar a pessoa idosa a se envolver nas mudanças de posição quando possível.
INTERVENÇÃO 51		Orientar o cuidador a manter a cama/leito da pessoa idosa limpo e com lençóis esticados para evitar tensionamento em feridas e cisalhamento no momento da mobilização.
INTERVENÇÃO 52		Orientar o cuidador a utilizar equipamentos apropriados para apoio de membros e proeminências ósseas, como coxins.
PERGUNTA 19	A pessoa idosa desenvolveu algum comportamento diferente durante esse distanciamento social?	Nenhuma/não
		Agitação
		Tristeza
		Agressividade
		Confusão
		Distúrbios de memória
		Distúrbios de orientação
		Distúrbios de comportamento
		Ansiedade
		Medo
INTERVENÇÃO 53		Orientar o paciente no tempo, espaço e pessoa.
INTERVENÇÃO 54		Fornecer um calendário.
INTERVENÇÃO 55		Estimular a cognição por meio de exercícios como pintura, desenho, quebra cabeça, cantar e ouvir música, utilizar televisão e rádio, colocar fotos de familiares próximos do paciente.
INTERVENÇÃO 56		Orientar quanto o ambiente em que a pessoa idosa dorme, escurecer o ambiente quando for o horário de dormir, ter uma ventilação adequada.
INTERVENÇÃO 57		Utilizar técnicas calmantes como músicas de preferência da pessoa idosa, tocá-lo e estar presente.
INTERVENÇÃO 58		Reconhecer os medos e os sentimentos do paciente.
INTERVENÇÃO 59		Permitir que a pessoa idosa mantenha alguns dos seus rituais para limitar a ansiedade.
INTERVENÇÃO 60		Informar a percepção do cuidador de maneira calma, tranquilizadora e não argumentativa.
INTERVENÇÃO 61		Responder ao tom, em vez de ao conteúdo, da alucinação ou delírio.
INTERVENÇÃO 62		Manter um ambiente bem iluminado que reduza contrastes agudos e sombras.
PERGUNTA 20	Houve uso de serviços de saúde?	Não
		Sim
DESFECHO		Continuou com o cuidado do SAD^‡^ após o término das ligações.
		Não quis mais receber a visita presencial do SAD^‡^ por causa da pandemia e nem as ligações da pesquisa (assinou o termo e não retirou a participação da pesquisa).
		Alta do SAD^‡^ após as ligações ou término das ligações.
		Internação
		Óbito
Motivo da internação ou óbito		Descritivo

^*^ IGM = Imunoglobulina M; ^†^IGG = Imunoglobulina G; ^‡^SAD = Serviço de Atenção Domiciliar; ^§^USF = Unidade de Saúde da Família

O instrumento da Figura 1 foi desenvolvido à luz da avaliação gerontológica e fundamentada na Teoria da Transição^12^ ^),(^ ^19^. Elaborou-se de forma sistematizada com o objetivo de desenvolver um plano adequado de cuidados e apoio na intervenção do estado geral da pessoa idosa e de seu cuidador, relacionando ao cuidado de ambos durante a investigação da síndrome gripal e isolamento social em tempos de COVID-19.

As perguntas e as intervenções do telecuidado foram elaboradas de acordo com as atribuições legais do profissional enfermeiro, baseadas nos diagnósticos de risco de tensão do papel do cuidador, risco de infecção e risco de síndrome do idoso frágil da Taxonomia da NANDA-I^14^. Associados esses diagnósticos, respectivamente, as intervenções do NIC^15^, partiram das classificações de melhora do enfrentamento; apoio ao cuidador; controle de infecção e proteção contra infecção; assistência do autocuidado, das atividades essenciais da vida diária, banho/higiene, vestir/arrumar-se, alimentação e transferência; estimulação cognitiva e facilitação da autorresponsabilidade e no Protocolo de Manejo Clínico do Coronavírus (COVID-19) na Atenção Primária à Saúde do Ministério da Saúde^20^. Quanto à comparação das avaliações e intervenções na saúde da pessoa idosa e de seu cuidador através da teleconsulta, utilizou-se os domínios de NANDA I e NIC.

Esses diagnósticos e intervenções foram escolhidos para promoção e prevenção da saúde da pessoa idosa e dos seus cuidados no isolamento social e visando ao controle da infecção por COVID-19. 

Quando identificada alguma situação que demandava urgência/emergência ou atendimento presencial, no momento da ligação telefônica, a equipe do SAD era acionada pela equipe de pesquisa e realizava o devido atendimento ou encaminhamento.

O instrumento foi validado por cinco especialistas em gerontologia, em março de 2020, e foi aplicado em sete pessoas idosas cadastradas no serviço de atendimento domiciliar que não foram incluídas no estudo antes do início da coleta de dados e, posteriormente, os dados preliminares foram publicados indicando a validação do instrumento^21^.

### Tratamento e análise dos dados

Os dados foram tabulados utilizando o *software Microsoft Excel* 2016. Em seguida, foram importados para o *software* estatístico *IBM-SPSS* (*Statistical Package for the Social Sciences*) versão 24, para devido tratamento analítico. Fez-se uso de estatísticas descritivas dispostas em forma de tabela, onde foram contabilizadas as frequências e porcentagens das variáveis em estudo. Após avaliações iniciais dos dados, aplicou-se métodos estatísticos para verificar associações e correlações entre as variáveis. Para avaliar o comportamento entre duas variáveis categóricas, fez-se uso do teste estatístico de Fisher (Teste de Associação), e, na avaliação de diferenças entre as médias, aplicou-se o teste de Kruskal-Wallis. 

### Aspectos éticos

A pesquisa foi aprovada pelo Comitê de Ética em Pesquisa com Seres Humanos da instituição-sede da pesquisa com o número do Certificado de Apresentação de Apreciação Ética (CAAE) 14354919.1.0000.5243 e 43682821.6.0000.5243. Celebrado acordo de cooperação técnica com o município a que o SAD estava vinculado.

Quanto ao recrutamento dos participantes, a equipe de pesquisa realizou o primeiro contato telefônico para explicar a pesquisa que seria realizada, obtendo, inicialmente, o consentimento verbal de participação por meio da leitura do Termo de Consentimento Livre e Esclarecido (TCLE) tanto para a pessoa idosa quanto para o cuidador. Na oportunidade, foi ressaltado o sigilo das informações. Posteriormente, quando da visita presencial pela equipe do SAD em parceria com a equipe de pesquisa, foi obtida a assinatura do Termo de Consentimento Livre e Esclarecido de todos os participantes. 

Não houve riscos diretos aos participantes visto que as intervenções de enfermagem, ofertadas através das ligações telefônicas, já faziam parte do escopo de orientações e dos cuidados de enfermagem já ofertados à população do SAD. Observou-se um leve desconforto que pode ter sido causado pelos incômodos relacionados às chamadas e às possíveis alterações emocionais com referência às perguntas e intervenções do questionário que foram minimizados por parte dos pesquisadores com escuta ativa e oferta de suporte psicológico ou médico da equipe multiprofissional do SAD. O contato telefônico dos pesquisadores foi disponibilizado para o caso de intercorrências, orientações e encaminhamentos. Foram ofertados cuidados de enfermagem relativos à promoção da saúde através das orientações quanto à higiene, aglomeração, respeito ao isolamento social e manutenção de melhores condições de vida na pandemia da COVID-19 com melhora da capacidade emocional e funcional. 

Os benefícios do estudo tratam da contribuição social, acadêmica e profissional para a consolidação de um cuidado de enfermagem inovador no órgão público, de baixo custo e fácil manejo, utilizada no apoio ao serviço das equipes de enfermagem, promovendo auxílio na promoção da saúde, adesão ao tratamento, conforto psicológico, rastreio e prevenção de complicações de saúde da pessoa idosa.

Após final do estudo, o grupo controle recebeu todas as ligações com as devidas intervenções de enfermagem no mesmo período.

## Resultados

O presente estudo contou com o total de 1691 ligações, dessas, 1515 ligações para os 131 participantes do grupo intervenção e 176 ligações para os 88 participantes do grupo controle, através da utilização dos instrumentos de ligação adequados para cada grupo. 

Os resultados da Tabela 1 detalham as características dos participantes do estudo, bem como a separação por grupo analisado (controle e intervenção). O teste Exato de Fisher foi aplicado para avaliar a distribuição percentual da característica em relação aos grupos analisados, ou seja, sua homogeneidade.

**Tabela 1:** Avaliação da homogeneidade da amostra de pessoas idosas e cuidadores. São Gonçalo, RJ, Brasil, 2020

Característica	Grupo				Total		P-valor*
	Controle		Intervenção				
	n	%	n	%	n	%	
**Sexo da pessoa idosa**							0,165
Masculino	44	50,0	52	39,7	96	43,8	
Feminino	44	50,0	79	60,3	123	56,2	>0,999
**Sexo do cuidador**							
Masculino	13	15,7	17	15,2	30	15,4	
Feminino	70	84,3	95	84,8	165	84,6	
**Idade (média)**							
Pessoa idosa	75,2		75,9				0,849
Cuidador	51,6		53,1				0,615
**Possui cuidador**							
Sim	83	94,3	112	85,5	195	89,0	0,048
Não	5	5,7	19	14,5	24	11,0	0,068
**Número de comorbidade nas pessoas idosas**							
0	0	0,0	4	3,1	4	1,8	
1	7	8,0	14	10,7	21	9,6	
2	21	23,9	37	28,2	58	26,5	
3	22	25,0	29	22,1	51	23,3	
4	13	14,8	28	21,4	41	18,7	
5	11	12,5	14	10,7	25	11,4	
6	6	6,8	4	3,1	10	4,6	
7	4	4,5	1	0,8	5	2,3	
8	3	3,4	0	0,0	3	1,4	
9	1	1,1	0	0,0	1	0,5	0,709
**Número de comorbidades nos cuidadores**							
0	44	50,0	70	53,4	114	52,1	
1	28	31,8	34	26,0	62	28,3	
2	15	17,0	23	17,6	38	17,4	
3	1	1,1	4	3,1	5	2,3	

*Teste Exato de Fisher, significativo se p-valor < 0,05

Dentre as características analisadas quanto à homogeneidade, a única que apresentou significância foi “Possui cuidador”, já que observadas as diferenças percentuais entre os grupos (p-valor = 0,048), indicando que o grupo controle teve maior percentual de pessoas idosas com cuidador (94,3%) em comparação à intervenção (85,5%).

Foi realizada avaliação da homogeneidade das variáveis quantitativas quanto a: idade, idade do cuidador, número de comorbidades das pessoas idosas e número de comorbidades dos cuidadores, através dos resumos estatísticos e aplicação do teste de Kruskal-Wallis, com o objetivo de avaliar se a distribuição dos valores entre o grupo controle e intervenção eram similares. Destaca-se que o teste de Kruskal-Wallis é significativo se o p-valor for < 0,05.

Nesse sentido, observou-se que apenas a comparação ‘Número de comorbidades nas pessoas idosas’ no grupo controle foi maior do que no grupo intervenção (p-valor = 0,023). As demais comparações não foram significativas. 

Em relação às avaliações da saúde da pessoa idosa e dos cuidadores no que diz respeito aos sinais e sintomas da COVID-19, a saber: falta de ar, tosse, cansaço, dores, febre, coriza e a ausência de paladar e/ou olfato, durante as ligações, foi também aplicado o teste de Kruskal-Wallis com significância se o p-valor for < 0,05. Nessa avaliação, não houve diferenças percentuais entre o grupo controle e intervenção. 

Quanto à comparação das avaliações da saúde do cuidador em relação aos grupos analisados, observou-se significância na opção “Nenhum sintoma” favorecendo ao grupo controle (p - valor < 0,001). Este último teve mais avaliações positivas do que o grupo intervenção.

Já na comparação das avaliações da saúde dos moradores em relação aos grupos analisados, o resultado mais positivo foi “sem sintomas” no grupo intervenção com (p-valor < 0,001). 

Na Tabela 2, pode-se observar o número médio de intervenções realizadas com a pessoa idosa e com o cuidador durante as ligações no grupo intervenção.

**Tabela 2:** Número médio de intervenções realizadas no grupo intervenção com as pessoas idosas e cuidadores. São Gonçalo, RJ, Brasil, 2020

Semana de ligação	Domínio 7 - Papéis e Relacionamento				Domínio 11 - Segurança/proteção				Domínio 1 - Promoção da saúde			
	Diagnóstico - Risco de Tensão do Papel do Cuidador				Diagnóstico - Risco de Contaminação				Diagnóstico - Risco de Síndrome do Idoso Frágil			
	IC* de 95%				IC* de 95%				IC* de 95%			
	Média	Inferior^†^	Superior^‡^	P-valor^§^	Média	Inferior^†^	Superior^‡^	P-valor^§^	Média	Inferior^†^	Superior^‡^	P-valor^§^
1	4,73	4,39	5,06	**<0,001**	8,97	8,67	9,27	**<0,001**	4,99	4,02	5,96	**<0,001**
2	2,07	1,82	2,32		3,19	2,67	3,72		0,26	0,1	0,42	
3	1,85	1,62	2,08		2,41	1,93	2,88		0,15	0,04	0,27	
4	1,68	1,45	1,91		2,02	1,62	2,42		0,14	0,02	0,26	
5	1,6	1,4	1,81		1,5	1,25	1,76		0,1	0	0,2	
6	1,5	1,32	1,68		1,57	1,25	1,89		0,13	0	0,25	
7	1,47	1,3	1,65		1,62	1,28	1,95		0,04	0	0,11	
8	1,45	1,29	1,61		1,58	1,23	1,94		0,07	0	0,16	
9	1,45	1,29	1,62		1,57	1,21	1,93		0,02	0	0,06	
10	1,48	1,3	1,66		1,73	1,34	2,13		0,01	0	0,03	
11	1,49	1,31	1,67		1,71	1,29	2,13		0,03	0	0,09	
12	1,56	1,34	1,78		1,83	1,38	2,27		0,03	0	0,09	
13	1,69	1,38	1,99		1,82	1,38	2,26		0,02	0	0,06	
14	1,63	1,36	1,89		1,81	1,34	2,27		0,02	0	0,07	

*IC = Intervalo de confiança; ^†^Inferior = Estatística; ^‡^Superior = Estatística; ^§^P-valor = Teste Exato de Fisher, significativo se p-valor < 0,05

Observa-se que na primeira ligação há uma maior necessidade de intervenções. Ao longo das ligações, a quantidade diminui e esse resultado foi significativo (p-valor < 0,001) para todos os quesitos estudados, mas principalmente para a promoção da saúde, que trata da fragilidade e essas estabilizam ao longo do tempo, enquanto a Segurança/proteção permaneceu como algo necessário e foi reforçada com a pandemia de COVID-19, associada à promoção do bem-estar do cuidador incluso no domínio Papéis e Relacionamento. 

Na Tabela 3, avalia-se a existência de diferença no percentual de respostas de cada desfecho em relação ao grupo controle e intervenção. Há diferença nos desfechos analisados (p-valor < 0,001). Observa-se que no desfecho de alta, o resultado foi percentualmente significativo no grupo intervenção. Então, dos casos que tiveram alta do SAD (36), quase 84% foram no grupo intervenção. Importante destacar que a alta do SAD foi realizada pela equipe do serviço conforme a pesquisa avançou e as avaliações do paciente foram positivas.

**Tabela 3:** Comparação entre desfecho do grupo controle e intervenção de pessoa idosa e cuidadores. São Gonçalo, RJ, Brasil, 2020

Desfecho	Grupo				Total		P-valor*
	Controle		Intervenção				
	n	%	n	%	n	%	
1 Continuou com o cuidado do SAD^†^ após o término das ligações	67	44,4	84	55,6	151	100	<0,001
2 Alta do SAD^†^ após as ligações ou término das ligações	6	16,7	30	83,3	36	100	
3 Internação	11	64,7	6	35,3	17	100	
4 Óbito	4	26,7	11	73,3	15	100	

*P-valor = Teste Exato de Fisher, significativo se p-valor < 0,05; ^†^SAD = Serviço de Atenção Domiciliar

Para ponderar esses resultados da melhor forma, aplicou-se o uso da Razão de Chances e os resultados foram comparados entre o desfecho “alta” com os demais desfechos. Nessa análise, observa-se que a chance de resultado de alta no grupo intervenção foi maior quando comparada com outros desfechos no grupo controle. Assim, ter resultado de “alta” tem quase nove vezes mais chance de acontecer no grupo intervenção do que no grupo controle. 

## Discussão

Diante do momento de transição na saúde vivenciado ao redor do mundo devido à pandemia de COVID-19, a consequente mudança nos modos de vida do binômio pessoa idosa e seus cuidadores atendidos pelo SAD proporcionou a possibilidade da utilização dos recursos tecnológicos mediados pela telenfermagem, no apoio à continuidade do cuidado das pessoas idosas e seus cuidadores assistidos pelo serviço. 

De forma inédita, o estudo foi desenvolvido no auge da pandemia de COVID-19, embasados na cientificidade da combinação da Teoria da Transição^12^ ^),(^ ^19^, do Sistema de Linguagem Padronizada NANDA I^14^ e NIC^15^ para orientação documental do instrumento de ligação e também a regulamentação da Resolução COFEN 696/2022^22^. A combinação da teoria de transição através dos padrões de respostas possibilitou o embasamento da coleta de dados dos indicadores do processo de cuidar da pessoa idosa e de seus cuidadores, quais sejam: sentir-se ligado com os profissionais, interagir mesmo que por telefone, estar situado e desenvolver confiança e *coping*.

Já as linguagens padronizadas das Taxonomias de NANDA I^14^ e NIC^15^ foram cruciais na identificação dos principais diagnósticos de Enfermagem como: Síndrome do Idoso Frágil, Risco de Tensão do papel do cuidador e Risco de Contaminação. A Taxonomia de NIC consistiu nas principais intervenções mediante os respectivos diagnósticos.

Estudos internacionais na década de 1990 já apontavam que aproximadamente metade de todos os pacientes que vão para os cuidados domiciliares precisam de continuidade dos cuidados de enfermagem qualificados. Em tais estudos, destaca-se que, no momento da alta hospitalar, os pacientes ainda estão muito debilitados e não recuperados o suficiente para aprender os procedimentos necessários de autocuidado^23^.

Com isso, surge o ambiente de atendimento domiciliar através da Tecnologia *TeleHomeCare*; isto é, os estudos também previram que esta tecnologia ajudaria a fornecer o nível necessário de atenção e serviços para exercer os cuidados domiciliares às pessoas idosas e seus cuidadores pelas enfermeiras registradas em atendimento domiciliar e proficientes no *TeleHomeCare*. Diante disso, os estudos já demonstravam que, nas situações de calamidade pública, a criatividade e a busca de estratégias de atendimento remoto ultrapassam os desafios impostos pelas medidas de distanciamento e passam ocupar a pauta dos sistemas de saúde nos diferentes contextos de cuidado^23^.

Considerando o estado de calamidade pública e a proposta do estudo, foi possível unir a equipe de pesquisa e a equipe multiprofissional do SAD através de treinamentos, associando o uso da tecnologia e colocando em prática o planejamento de cuidados de enfermagem, contidos nas cinco etapas ininterruptas do Processo de Enfermagem sistematizado e composto no instrumento de ligação. A telenfermagem permitiu fornecer uma assistência de apoio e de continuidade inovadoras às pessoas idosas e aos cuidadores que estavam internados no domicílio nesse momento de isolamento social e que não tinham acesso aos serviços específicos de saúde. 

Vale ressaltar que os profissionais de saúde precisaram ser qualificados a desenvolver habilidades/competências inovadoras para garantir a qualidade, a segurança e a eficiência do cuidado por meio da tecnologia. Isso justifica a criação do instrumento de ligações pautado na transição do cuidado presencial para o remoto durante a pandemia COVID-19^24^. 

Assim como as demais regulamentações de acesso aos dados da sociedade, a telenfermagem também deve ser previamente consentida pelo paciente ou por seu representante legal, e sua realização é de responsabilidade do enfermeiro. O registro da teleconsulta foi obrigatório e pautado no processo de enfermagem (consulta de enfermagem, diagnóstico, planejamento, intervenção e avaliação), para organização e orientação do cuidado às pessoas idosas e seus cuidadores. Desse modo, nas ligações, as pessoas idosas e seus cuidadores do SAD foram abordados quanto à coleta de dados embasada na combinação da teoria de transição, os diagnósticos foram extraídos dos domínios do NANDA-I^14^: papéis e relacionamentos familiares, segurança/proteção e promoção à saúde.

No domínio I promoção da Saúde, foi extraído o diagnóstico Risco da Síndrome do idoso frágil, pois, apesar de não haver uma definição consensual, a fragilidade pode ser compreendida como uma síndrome multidimensional, que envolve a interação complexa de fatores biológicos, psicológicos e sociais. Essa interação culmina numa maior vulnerabilidade e está associada aos riscos de desfechos clínicos como o declínio funcional, quedas, desnutrição, hospitalização e morte. Logo, um plano de cuidados de enfermagem quanto a esses sinais e sintomas foram essenciais. As intervenções de NIC foram relacionadas ao diagnóstico de enfermagem quanto à prevenção das complicações agudas, à orientação para a adoção de uma alimentação equilibrada, cuidados pessoais e para manter-se ativo^25^.

Já no Domínio 7, Papéis e Relacionamentos, a justificativa também se deu mediante a melhor cobertura assistencial à pessoa idosa e seus cuidadores na pandemia COVID-19. Nesse domínio, foi devido a possível sobrecarga física e emocional do cuidador da pessoa idosa. As intervenções de NIC foram justificadas com o objetivo de minimizar os efeitos deletérios da tensão do papel do cuidador através das orientações de enfermagem dispostas no instrumento de ligação. No domínio 11, Segurança e Proteção, destacou-se o Risco de Infeção durante a pandemia COVID-19. As intervenções foram também guiadas de forma sistematizada através do instrumento de ligação. 

Durante as ligações, identificou-se e confirmou-se que as pessoas idosas e seus cuidadores possuíam doenças crônicas e eram acompanhados pelo SAD e pela Rede de Assistência à Saúde do município sem qualquer regularidade. Estudos corroboram que, com o agravo da Pandemia COVID-19, o esforço do cuidado, de um modo geral, aumentou significativamente e isso se refletiu na saúde física e mental da população, em especial das mulheres em que quase uma em cada duas tem comorbidades crônicas que se agravaram na pandemia. E, consecutivamente os sentimentos de isolamento, tristeza e ansiedade por parte desse binômio - pessoas idosas e seus cuidadores - também foram aumentados^26^ ^)-(^ ^27^. 

Tal dado vem ao encontro do presente estudo, considerando o percentual alto de pessoas idosas que possuíam cuidadoras do gênero feminino, sendo essa recorrência maior em comparação ao gênero masculino, conforme pôde ser visto na Tabela 1. Houve identificação também do gênero feminino para cuidadoras que não recebiam remuneração, o que vem ao encontro de estudos sobre o cuidado prestado às pessoas idosas^28^.

Nas ligações realizadas para a pessoa idosa e seus cuidadores, foi possível observar a necessidade um olhar diferenciado do Estado, da sociedade e das famílias quanto à organização e um planejamento familiar. Estudos já narram a importância da inclusão do cuidador não só como executor das ações planejadas, mas como sujeito principal na promoção e na qualidade do cuidado, quando a pessoa idosa perde a condição do autocuidado. 

A visibilidade do papel do cuidador foi relevante no estudo já que, através dele, foi possível se levar assistência à saúde ao binômio pessoa idosa e cuidador. Políticas públicas de saúde são necessárias na cobertura do cuidado integral à saúde da pessoa idosa e seus cuidadores, afinal o cuidador está na “linha de frente anônima” no cuidado à pessoa idosa, não só durante a pandemia COVID-19, mas como requisito para a adesão da pessoa idosa no Serviço de Atenção Domiciliar^29^.

A telenfermagem trouxe à tona um número significativo de intervenções na primeira ligação e se manteve, durante as quatro primeiras semanas, mediante o pânico que se instalou nos cidadãos, mediante as incertezas e óbitos que vinham sendo provocados pela pandemia. O estado pandêmico não viabilizava a visitais presencias da equipe do SAD e a assistência à saúde da população de um modo geral ficou prejudicada.

Nesse sentido, a telenfermagem como apoio nas intervenções de enfermagem na continuidade do cuidado à pessoa idosa e seus cuidadores foi inovadora e essencial para sanar as dúvidas dos pacientes e minorar o medo da interação que, de acordo com as notícias veiculadas na mídia eram todas preditoras de óbitos. Nesse episódio, foi possível a equipe de ligação desenvolver suas ações de enfermagem com maestria e adquirir a confiança da pessoa idosa e seus cuidadores assistidos pelo SAD. 

Depois das quatro semanas, pode-se observar que a quantidade de ligações diminuiu no quesito do diagnóstico do Risco da Síndrome do Idoso Frágil e se equiparou às demais intervenções de Risco de Contaminação e Risco de Tensão do Papel do Cuidador, resultado esse positivo (p-valor < 0,001). 

As recomendações de segurança com pessoas idosas foram muito além das que já eram realizadas diariamente pelo cuidador. Fez-se necessário a intensificação dos cuidados e a atenção para ações preventivas ligadas diretamente ao comportamento e à higiene, já que o momento era de muita solidão e dúvidas quanto à doença^19^ ^),(^ ^22^ ^),(^ ^30^ ^)-(^ ^31^. 

Outros resultados se assemelham aos estudos nacionais e internacionais, sobretudo, no que diz respeito à pessoa idosa e seus cuidadores continuarem no SAD, receberem alta, serem internados ou irem a óbito após as ligações ou término das ligações. Houve um destaque de 83.3% para o grupo intervenção, pois no auge da COVID-19, as internações se destinavam aos casos de maior gravidade como a Síndrome da insuficiência respiratória aguda, o que culminou no aumento de alta dos pacientes de toda a Rede de Assistência à Saúde e também nos aderidos pelo SAD^26^ ^)-(^ ^27^. É importante ressaltar que a alta da pessoa idosa e seus cuidadores assistidos pelo SAD, durante a pesquisa, foi possível mediante as informações atualizadas que a equipe de pesquisa transmitia à equipe multiprofissional do SAD, sendo que esta última prestava também a continuidade do cuidado através da tecnologia até a possível alta.

Diante dos significativos resultados, foi notória a importância dos profissionais de saúde na minimização dos efeitos causados pela calamidade pública de saúde na pandemia COVID-19, especialmente em relação aos trabalhadores de enfermagem, protagonistas no controle da transmissão dessa doença, tanto na linha de frente quanto nas intervenções de apoio à continuidade do cuidado a pessoa idosa e seus cuidadores por meio da telenfermagem. 

Portanto, os resultados mostraram que a telenfermagem realizada pelo enfermeiro como tecnologia de apoio na transição do cuidado às pessoas idosas e seus cuidadores na pandemia de COVID-19 obteve um índice de confiança de 95% e significância (p-valor < 0,001) em todos os domínios e diagnósticos estudados (Papéis e Relacionamento - Risco de Tensão do Papel do Cuidador; Segurança e Proteção - Risco de Contaminação e Promoção da saúde - Risco de Síndrome do Idoso Frágil). Destaca-se a importância da telenfermagem nas intervenções realizadas pela equipe de enfermeiros, com resultados positivos na promoção da saúde da pessoa idosa e seus cuidadores.

Em termos de informações, a salvaguarda das informações e dos registros obtidos durante a telenfermagem é de responsabilidade do profissional que a executou e/ou da instituição de saúde vinculada. A prática da telenfermagem requer adequações das partes envolvidas - enfermeiro e paciente - que vão além de infraestrutura e conectividade^21^. Nesse sentido, o enfermeiro encaminha à informação a equipe do SAD para atestar o óbito.

O “fazer saúde” é realizado pela equipe de enfermagem ou equipe multiprofissional nos mais diversos contextos, de forma que cada categoria profissional possui seu arcabouço de saberes e práticas que objetivam o diagnóstico, a prevenção e a recuperação de doenças e agravos que acometem o indivíduo, a família e a coletividade. É importante saber que a telenfermagem deve ser desenvolvida por profissional de enfermagem com competência técnica e autonomia e que saiba distinguir uma prática avançada de enfermagem à de uma generalista^32^. 

O suporte da teoria das transições foi essencial para fundamentar os objetivos terapêuticos ao apontar os resultados de indicadores do processo de cuidar: sentir-se ligado, interagir, estar situado e desenvolver confiança e *coping*. A maestria foi contemplada, pois, segundo a teoria, na ocorrência da integração fluida entre a equipe de enfermeiros no apoio ao cuidado do binômio pessoa idosa e cuidadores, essenciais, diante de situações de crise como a da pandemia, o cuidado à pessoa idosa tornou-se redobrado por ser o grupo mais susceptível a complicações da COVID-19. A comprovação disso foi a redução no número de intervenções após as primeiras semanas.

No que tange às limitações do estudo, pode-se dizer que no auge da pandemia o acesso aos dados da pessoa idosa e seus cuidadores cadastrados no SAD se encontravam desatualizados, o que impossibilitou a randomização do estudo. Essas limitações levaram a equipe de pesquisa a auxiliar a equipe do SAD na atualização dos cadastros das pessoas idosas e de seus cuidadores aderidos ao SAD, concomitante a realização da pesquisa. A escassez de dados a respeito da COVID-19, também foi uma limitação. 

Já em relação às implicações para o avanço do conhecimento científico para a área da saúde e enfermagem, observou-se que a telenfermagem pode ser responsável por implicações positivas e negativas quando desenvolvidas por leigos como um *call center*. Os cuidados de enfermagem através da telenfermagem podem ser realizados em toda a rede pública e privada de assistência à saúde da pessoa idosa, seus cuidadores e demais cidadãos que necessitem das intervenções de enfermagem. A tecnologia de apoio aos enfermeiros demanda competências técnicas e científicas necessárias para a tomada de decisão clínica assertiva. Os instrumentos de ligação pautados em evidências científicas são úteis para direcionar o planejamento do cuidado à pessoa idosa e seus cuidadores no curso da pandemia COVID-19. As ligações devem estar pautadas de forma clara na doença e no papel do enfermeiro quanto ao cuidado longitudinal ao binômio pessoa idosa e seus cuidadores assistidos pelo Sistema de Atenção Domiciliar ou demais nichos da saúde.

A telenfermagem apesar de já existir no mundo, só na pandemia COVID-19, foi regulamentada no Brasil pelo Sistema Cofen/Corens de forma emergencial para o apoio dos enfermeiros nas tomadas de decisões clínicas. Diante disso, é fundamental que sejam desenvolvidas novas pesquisas de efetividade da telenfermagem como apoio na continuidade do cuidado à saúde integral da saúde da pessoa idosa e seus cuidadores. O SAD, por ser um equipamento de assistência multidisciplinar para toda a equipe de saúde, possibilitaria um acompanhamento à saúde dos seus cadastrados com diversas possibilidades de intervenções positivas de apoio à continuidade do cuidado longitudinal^(33)-(34)^. 

## Conclusão

Os resultados demonstraram a relevância do uso da cientificidade quanto à combinação da teoria de transição com a taxonomia de diagnóstico e intervenções para a continuidade do planejamento de cuidados à pessoa idosa e seus cuidadores assistidos pelo SAD, através da telenfermagem, com a chance de alta nove vezes maior quando se recebe o cuidado por telefone. Os diagnósticos de enfermagem evidenciados neste artigo como Risco de Tensão do Papel do Cuidador; Risco de Síndrome do Idoso Frágil e Risco de Contaminação, além das intervenções contidas no instrumento de ligação foram eficientes e essenciais, pois permitiram atender ao objetivo do estudo no desenvolvimento da telenfermagem como apoio à continuidade do cuidado presencial para o remoto a pessoa idosa e seus cuidadores, focados na prevenção e promoção à saúde no contexto pandêmico. Assim, o apoio através da telenfermagem também permitiu à equipe multiprofissional do SAD uma análise precisa quanto à alta com segurança dos pacientes aderidos ao serviço, encaminhando-os para a rede de Atenção Primaria à saúde para atendimentos de baixa complexidade.

# Translation: Teleenfermería con personas mayores en el servicio de atención domiciliaria durante la pandemia de COVID-19: estudio cuasiexperimental*

**DOI:** 10.1590/1518-8345.7138.4319

**Authors:** Maria Auxiliadora Rodrigues, Rosimere Ferreira Santana, Ana Beatriz Serra Hercules, Patricia de Fátima Augusto Barros, Clelia Barboza Lima

**Affiliations:** 1 Universidade Federal Fluminense, Escola de Enfermagem Aurora de Afonso Costa, Niterói, RJ, Brasil.; 2 University of Central Florida, Orlando, FL, Estados Unidos de América.

**Keywords:** Teleenfermería, COVID-19, Servicios de Atención de Salud a Domicilio, Cuidadores, Atención de Enfermería, Tecnología para la Salud

## Abstract

**Objetivo:**

evaluar la teleenfermería como tecnología de apoyo en la transición del cuidado a las personas mayores y sus cuidadores en el contexto de la atención domiciliaria durante la pandemia de COVID-19.

**Método:**

estudio cuasi experimental tipo antes-después, no aleatorizado, con 219 personas mayores y con cuidadores del servicio de atención domiciliaria, divididos en 131 en el grupo de intervención y 88 en el grupo de control. Se realizó tratamiento analítico, estadísticas descriptivas e inferenciales.

**Resultados:**

se realizaron 1691 llamadas, siendo 1515 para el grupo de intervención y 176 para el control. Se observó que en la primera llamada hay una mayor necesidad de intervenciones para la promoción de la salud y que esa cantidad disminuye a lo largo de las llamadas con resultado significativo (p-valor < 0,001). Los desenlaces analizados fueron hospitalización, fallecimiento, alta o continuación del servicio de atención domiciliaria y se observó que la probabilidad de alta del servicio fue nueve veces mayor en el grupo de intervención. La continuidad del cuidado del servicio de atención domiciliaria y alta después del término de las llamadas también fueron significativos (p-valor < 0,001).

**Conclusión:**

la teleenfermería fue una tecnología de apoyo al cuidado, principalmente para la promoción de la salud y en la alta del servicio de atención domiciliaria.

## Destacados

**(1)** La teleenfermería contribuye en el proceso de transición del cuidado en pandemias y a la persona mayor.

**(2)** Alta del servicio de atención domiciliaria nueve veces mayor en el grupo de intervención.

**(3)** Disminución del número de intervenciones a lo largo de la aplicación de la teleenfermería.

**(4)** Tecnología accesible y de fácil utilización aplicada tanto para la persona mayor como para el cuidador.

**(5)** Instrumento de conexión/intervención fundamentado en teoría y proceso de enfermería.

## Introducción

Con el agravamiento de la pandemia, debido a la diseminación de la enfermedad *Coronavirus Disease-19* (COVID-19), que se desarrolló de manera tan rápida y dramática, los servicios de salud entraron en colapso y no pudieron responder rápidamente a la rutina del proceso de trabajo. El inicio de este caos en la salud se dio en diciembre de 2019, a partir de muestras de lavado broncoalveolar obtenidas de pacientes con neumonía de causa desconocida en Wuhan, provincia de Hubei, China, cuando se descubrió el betacoronavirus denominado *Severe Acute Respiratory Syndrome Coronavirus-2* (Sars-Cov-2)^1^ ^)-(^ ^3^.

En ese período, hubo una mayor preocupación por la población de personas mayores, que representa 31,2 millones de personas en Brasil, correspondiendo a casi el 15% de la población total^4^, y que, debido al proceso de envejecimiento, posee un deterioro del sistema inmunológico, principalmente al asociarse a las enfermedades crónicas también relativas a la edad, puede promover un aumento de la vulnerabilidad a las formas graves de COVID-19, incluso, pudiendo evolucionar hacia la muerte^5^. Esto se comprobó cuando el primer fallecimiento confirmado fue de un hombre de 62 años, diagnosticado con diabetes mellitus y hipertensión arterial^6^ y, también, a partir de estudios que demostraron que la mayor letalidad en Brasil estaba entre la franja etaria de más de 60 años, representando el 69,3% de los fallecimientos confirmados en 2020. De estos fallecimientos, el 64% presentaban al menos un factor de riesgo como cardiopatía, diabetes mellitus, enfermedad renal, entre otras. La junta de la Organización Mundial de la Salud y China presentaron estudios sobre las mismas comorbilidades en la población de personas mayores, lo que demostró que son indicadores de mayor riesgo para mortalidad con la COVID-19^5^ ^),(^ ^7^.

Con los profesionales de salud de primera línea afectados de forma significativa por esta nueva enfermedad, se abrió una oportunidad para realizar atenciones de salud a larga distancia, considerando el distanciamiento como medida de protección debido al alto grado de transmisión y contaminación del virus Sars-Cov-2, además de la sobrecarga de la red de salud y la prioridad de atención hospitalaria a los casos graves. Dicha estrategia es conocida como telemedicina desde los años 90 y se caracteriza por el uso de las tecnologías de la información y comunicación para la prestación de cuidados en salud. El concepto aplicado a la enfermería se llama teleenfermería y tiene amplia aplicación y eficacia en los servicios de atención a la salud, gestión, asistencia, enseñanza e investigación, además de las ventajas de ampliar la cobertura de salud y de reducir costos. Así, con el avance rápido de la pandemia, los órganos nacionales fueron impulsados a transformar el servicio de telemedicina y teleenfermería - de un servicio de apoyo para la principal estrategia de atención al paciente^8^ ^)-(^ ^11^.

Tal escenario se fundamenta y se justifica en la Teoría de la Transición, que consiste en el “pasaje de un estado, condición o lugar, a otro”. Así, ella se inicia mediante eventos incontrolables^12^. En este contexto, la pandemia de COVID-19 forzó el aislamiento físico y social en el mundo, presentándose como uno de esos eventos incontrolables, de modo a proporcionar a las personas mayores una transición en el cuidado, más específicamente, del cuidado presencial para el remoto. La teoría ejerce una fuerte influencia en la intervención de enfermería, a partir de un cuidado transicional con una relación entre persona mayor-cuidador-enfermero planeado antes de situaciones de cambios en la vida, con estrategias en el proceso del cuidado de transición que mejore la calidad de vida de las personas, disminuyendo el riesgo potencial que la experiencia de transición puede proporcionar. Las fases del proceso de transición se dividieron en fase de entrada, con el inicio de la pandemia y del contacto telefónico; pasaje por el proceso de intervención telefónica; y salida, identificado como desenlace en este estudio. El desenlace puede ser positivo - que sería la no contaminación por COVID-19 y la continuidad del cuidado de comorbilidades crónicas - o negativo - que sería la contaminación o discontinuidad del cuidado, provocando internaciones o fallecimientos^12^ ^)-(^ ^13^.

Se entiende que la transición es esencialmente positiva, pues en el proceso de ella se obtiene mayor madurez y estabilidad y cuando aplicado a los cuidados de enfermería realizados por teléfono, tienden a ser aprehendidos y reproducidos, ya que no hay un intermediario para realizarlo, solo el cuidador. Además, caso esa transición cause ansiedad, desorientación, irritabilidad y estrés, el teléfono es una vía fácil e inmediata de comunicación para auxiliar en el proceso y obtener éxito y *coping*, conforme describe la teoría de la transición^12^ ^)-(^ ^13^.

Siendo así, para entonces actuar en la transición del cuidado presencial al remoto con las intervenciones de enfermería orientadas específicamente para las personas mayores y cuidadores, se utilizaron los diagnósticos de enfermería de la Taxonomía de NANDA-I^14^, basados en los diagnósticos de riesgo de tensión del papel del cuidador, riesgo de infección, riesgo de síndrome del anciano frágil. Asociados a los diagnósticos de enfermería, las intervenciones del NIC^15^, respectivamente a los diagnósticos NANDA-I partieron de las clasificaciones de mejora del enfrentamiento; apoyo al cuidador; control de infección y protección contra infección; asistencia del autocuidado, de las actividades esenciales de la vida diaria, baño/higiene, vestir/arreglarse, alimentación y transferencia; estimulación cognitiva y facilitación de la autorresponsabilidad.

De esta forma, considerando el contexto pandémico, con las personas mayores como grupo de riesgo, consecuentemente estando en situación de vulnerabilidad, con cuidadores sobrecargados, insertados en el sistema de salud en colapso, así como teniendo en cuenta el avance de la teleenfermería en la transición del cuidado presencial al remoto, este estudio tuvo por objetivo evaluar la teleenfermería como tecnología de apoyo en la transición del cuidado a las personas mayores y sus cuidadores en el contexto de la atención domiciliaria en la pandemia COVID-19. Los desenlaces del objetivo del estudio incluyen alta del servicio de atención domiciliaria (SAD), que significa continuidad del cuidado en la atención básica, internación o fallecimiento.

## Método

### Tipo del estudio

Se trata de un extracto de la tesis doctoral titulada “Teleenfermería en la continuidad del cuidado a la persona mayor y cuidador en el SAD durante la COVID-19: estudio cuasi experimental” para evaluar la teleenfermería como tecnología de apoyo en la transición del cuidado a la persona mayor y su cuidador en la pandemia de COVID-19. Estudio cuasi experimental del tipo antes y después, no aleatorizado. Para la construcción del método, se utilizó el CONSORT.

### Lugar del estudio

La investigación se desarrolló en el SAD en São Gonçalo, RJ, Brasil.

### Período

El estudio se realizó en el período de marzo a mayo de 2020.

### Población

La población del estudio estuvo compuesta por personas mayores registradas en el SAD y sus cuidadores.

Dado el acuerdo de cooperación establecido con el SAD, se proporcionó una lista telefónica con todos los pacientes registrados y atendidos por ese servicio, que comprendían 392 personas, incluyendo niños, adultos y personas mayores.

### Criterios de selección

Los criterios de inclusión fueron estar registrados en el SAD; tener edad igual o superior a 60 años. Si poseen cuidador: ser el principal cuidador de la persona mayor acompañada por el SAD; aceptar participar en la investigación; ser capaz de responder a los cuestionamientos realizados por la investigadora de forma coherente y poseer teléfono fijo o móvil.

Los criterios de discontinuidad aplicados: no responder a la primera llamada tras tres intentos; no atender, como mínimo, al 25% de las llamadas telefónicas después del inicio de la teleconsulta de enfermería.

Cabe destacar que uno de los criterios para estar registrado a un servicio de atención domiciliaria del Sistema Único de Salud es tener un cuidador^16^, por lo tanto, todas las personas registradas poseían cuidadores vinculados en la ficha catastral, pudiendo ser familiar o profesional, pero en la práctica no todos estaban con los cuidadores presentes en el momento de la llamada o omitieron la información al servicio para que pudieran recibir el cuidado en salud incluso sin tener el cuidador, siendo así el instrumento fue aplicado de acuerdo con quien atendía la llamada.

### Definición de la muestra y recolección de datos

El tamaño de la muestra fue determinado con la ejecución de metodología muestral que estimó la proporción de resultados de los desenlaces del servicio de atención domiciliaria en dos poblaciones, en este caso: control e intervención. En este sentido, sea *p*_*1*_ la proporción de altas del servicio de atención domiciliaria en el grupo control y sea *p*_*2*_ la proporción de casos en el grupo intervención, es necesario medir la diferencia *p*_*1*_ - *p*_*2*_ , también conocida como diferencia de riesgos^17^. Para el cálculo fueron necesarias informaciones de estudios pilotos y de informaciones previas de estudios ya conocidos o publicados^18^. En el estudio piloto, encontramos los siguientes valores *p*_*1*_ =0.05 y *p*_*2*_ =0.20, aproximadamente. Se supuso, entonces, que en el grupo intervención la proporción de alta sería el cuádruple del grupo control. Considerando estos valores y una variación del nivel de confianza del test de 95%, se obtuvo que la muestra mínima sería de 188 personas mayores, quedando evidente que la población del presente estudio (219 participantes) es satisfactoria.

La lista telefónica proporcionada poseía nombre en orden alfabético y edad de las 392 personas registradas en el SAD. Estas fueron divididas en forma de sorteo aleatorio en dos sobres distintos, nombrados grupo control y grupo intervención, que contenían 196 nombres cada uno. Se abrieron los sobres y, tras la aplicación de los criterios de inclusión, se identificaron 302 personas mayores elegibles para el estudio, siendo 90 excluidas, 73 del grupo control y 17 del grupo intervención. Tras la primera llamada, 10 personas mayores del grupo control y 4 del grupo intervención no atendieron la primera llamada tras tres intentos en días y horarios diferentes, considerándose estas como pérdidas de participantes, resultando en 288 incluidos en el estudio, siendo 113 en el grupo control y 175 en el grupo intervención.

En el transcurso del estudio, 69 personas mayores fueron excluidas por no atender el 25% de las llamadas telefónicas, siendo 44 del grupo intervención y 25 del control, obteniéndose una muestra final de 219 personas mayores, siendo 131 en el grupo intervención, 88 en el control (Figura 1).

**Figura 1:** Flujograma de participantes y protocolo de intervención de la investigación aplicado a los grupos control e intervención *SAD = Servicio de Atención Domiciliaria

### Instrumentos

El equipo de investigación, formado por la investigadora principal, además de dos enfermeras y dos estudiantes de enfermería, creó dos instrumentos de llamada para ser utilizados en el contacto telefónico, uno para el grupo intervención y otro para el grupo control (Figura 2), que sirvieron para guiar las llamadas telefónicas realizadas.

**Figura 2:** Instrumento de investigación del grupo control e intervención para persona mayor y cuidadores

Instrumento de Control		
NÚMERO	PREGUNTA	LEYENDA
CUIDADOR		Autocuidado/uno mismo
		No formal
		Formal
TIPO DE CUIDADOR		Autocuidado/uno mismo
		Cónyuge
		Hijo(a)
		Padre/madre
		Nieto(a)
		Hermano(a)
		Sobrino(a)
		Primo(a)
		Nuera
		Vecino(a)
		Cuidador formal
ESTADO DE LA PERSONA MAYOR - ¿Cómo está usted? ¿Y la persona mayor? ¿Cuántas personas hay en la casa? ¿Cuántas personas hay en el terreno?		Descriptiva
RESIDENTES DE LA CASA - ¿Cuántos residentes hay en la casa?		Número de residentes
PREGUNTA 1	¿Cómo está el cuidado de la persona mayor en casa en tiempos de distanciamiento social para la prevención del coronavirus?	Tranquilo
		Puede realizar todas las actividades planificadas en el día
		Cansado
		Ajetreado
		No puede realizar las actividades planificadas en el día
		Estrés mental
		Sin comida o suministros
		Sin medicación
PREGUNTA 2	¿Hubo algún cambio en la rutina para adaptarse al cuidado de la persona mayor? ¿Hubo aumento en las demandas de cuidado durante la pandemia?	No
		SÍ
PREGUNTA 3	¿La persona mayor está aislada?	No
		Sí
PREGUNTA 4	¿Alguien en la casa trabaja? ¿Y sale de casa para trabajar?	Nadie trabaja
		Trabaja en Actividades Esenciales y sale de casa
		Trabaja en Actividades No Esenciales y sale de casa
		Trabaja en Actividades No Esenciales y está en *home office*
PREGUNTA 5	¿Cómo está la salud de la persona mayor?	Ningún síntoma/todos bien
		Falta de aire
		Tos
		Cansancio
		Dolores
		Fiebre
		Congestión nasal
		Sin olfato y/o gusto
PREGUNTA 5.1	¿Cuánto tiempo?	Número en días
PREGUNTA 6	¿La persona mayor se hizo la prueba rápida de COVID?	No
		Sí
PREGUNTA 6.1	Si es así, ¿cuál fue el resultado?	Negativo
		Positivo
PREGUNTA 6.2	Si es positivo, ¿cuál fue el resultado?	No se aplica
		IGM* positivo
		IGG^†^ positivo
PREGUNTA 7	¿El cuidador está aislado?	No
		Sí
		No se aplica
PREGUNTA 8	¿Cómo está la salud del cuidador?	Ningún síntoma/todos bien
		Falta de aire
		Tos
		Cansancio
		Dolores
		Fiebre
		Congestión nasal
		Sin olfato y/o gusto
		No se aplica
PREGUNTA 8.1	¿Cuánto tiempo?	Número en días
PREGUNTA 9	¿El cuidador se hizo la prueba rápida de COVID?	No
		Sí
		No se aplica
PREGUNTA 9.1	Si es así, ¿cuál fue el resultado?	No se hizo la prueba/no se aplica
		Negativo
		Positivo
PREGUNTA 9.2	Si es positivo, ¿cuál fue el resultado?	No se aplica
		IGM* positivo
		IGG^†^ positivo
PREGUNTA 10	¿Cómo está la salud de las otras personas de la casa?	Ningún síntoma/todos bien
		Falta de aire
		Tos
		Cansancio
		Dolores
		Fiebre
		Congestión nasal
		Sin olfato y/o gusto
		No se aplica
PREGUNTA 10.1	¿Cuánto tiempo?	Número en días
PREGUNTA 11	¿Alguien más en la casa se hizo la prueba rápida?	No
		Sí
PREGUNTA 11.1	Si es así, ¿quién? *Considerar personas además de la persona mayor y el cuidador. Ejemplo: si el cuidador es el cónyuge, esta pregunta no debe ser considerada, pero si el cuidador es el hijo y el marido se hizo la prueba, esta pregunta debe ser respondida.*	No se aplica
		Cónyuge de la persona mayor
		Hijo(a) de la persona mayor
		Nieto(a) de la persona mayor
		Padres de la persona mayor
		Hermano(a) de la persona mayor
		Cónyuge del cuidador
PREGUNTA 11.2	Si es así, ¿cuál fue el resultado?	No se hizo la prueba
		Negativo
		Positivo
PREGUNTA 11.3	Si es positivo, ¿cuál fue el resultado?	No se aplica
		IGM* positivo
		IGG^†^ positivo
PREGUNTA 12	¿La persona mayor ya fue vacunada contra la gripe?	No
		Sí
PREGUNTA 12.1	¿Quién la vacunó?	SAD^‡^
		USF^§^
PREGUNTA 13	¿El cuidador ya fue vacunado contra la gripe?	0 No se aplica
		1 No
		2 Sí
PREGUNTA 13.1	¿Quién la vacunó?	No se aplica
		SAD^‡^
		USF^§^
PREGUNTA 14	¿Ha recibido visitas del SAD? ¿Con qué frecuencia?	No
		Semanalmente
		Cada 15 días
		Mensual
PREGUNTA 15	¿Hubo uso de servicios de salud?	No
		Sí
DESENLACE		Continuó con el cuidado del SAD^‡^ después del término de las llamadas.
		No quiso seguir recibiendo la visita presencial del SAD^‡^ por causa de la pandemia ni las llamadas de la investigación (firmó el término y no retiró la participación en la investigación).
		Alta del SAD^‡^ después de las llamadas o término de las llamadas.
		Internación
		Fallecimiento
Motivo de la internación o fallecimiento		Descriptivo
**Instrumento de Intervención**		
**COLUMNA**	**PREGUNTA**	**LEYENDA**
CUIDADOR		Autocuidado/uno mismo
		No formal
		Formal
TIPO DE CUIDADOR		Autocuidado/uno mismo
		Cónyuge
		Hijo(a)
		Padre/madre
		Nieto(a)
		Hermano(a)
		Sobrino(a)
		Primo(a)
		Nuera
		Vecino(a)
		Cuidador formal
ESTADO DE LA PERSONA MAYOR - ¿Cómo está usted? ¿Y la persona mayor? ¿Cuántas personas hay en la casa? ¿Cuántas personas hay en el terreno?		Descriptivo
ESTADO DEL CUIDADOR		Descriptivo
RESIDENTES DE LA CASA - ¿Cuántos residentes hay en la casa?		Número de residentes
PREGUNTA 1	¿Cómo está el cuidado de la persona mayor en casa en tiempos de distanciamiento social para la prevención del coronavirus?	Tranquilo
		Puede realizar todas las actividades planificadas en el día
		Cansado
		Ajetreado
		No puede realizar las actividades planificadas en el día
		Estrés mental
		Sin comida o suministros
		Sin medicación
INTERVENCIÓN 1		Usar un enfoque calmado y tranquilizador.
INTERVENCIÓN 2		Fomentar la verbalización de sentimientos, percepciones y miedos.
INTERVENCIÓN 3		Proporcionar al cuidador información realista sobre los aspectos del cuidado a la persona mayor.
INTERVENCIÓN 4		Evaluar la posibilidad de turnarse en los cuidados.
INTERVENCIÓN 5		Fomentar el diálogo del cuidador con familiares sobre los cuidados.
PREGUNTA 1.1	Si la respuesta es *Cansado, Ajetreado o No puede realizar las actividades planificadas en el día:* ¿Por qué dice eso?	Descriptivo
PREGUNTA 2	¿Cuál es su sentimiento relacionado al cuidado prestado a la persona mayor en este tiempo de pandemia?	Calmo/tranquilo
		Satisfecho
		Triste
		Cansado
		Sobrecargado
		Agotado
		Angustiado
		Estresado
		Inseguridad
PREGUNTA 2.1	¿Con qué frecuencia?	No/Nunca
		Raramente
		A veces
		Casi siempre
		Siempre
INTERVENCIÓN 6		Fomentar la participación de la familia en línea, según corresponda.
INTERVENCIÓN 7		Solicitar servicios de otros profesionales de salud para el paciente, según corresponda.
INTERVENCIÓN 8		Actuar en favor del cuidador cuando la sobrecarga se vuelva evidente/Explicar sobre el funcionamiento de la red de asistencia de salud, como la llamada al núcleo del equipo multiprofesional de apoyo que ofrecerá soporte al cuidador y a la familia en cuestiones sociales y psicológicas.
INTERVENCIÓN 9		Fomentar la realización de actividades de ocio en el domicilio (se puede enviar material por *Web Whats* Comercial).
PREGUNTA 3	¿La persona mayor está aislada?	No
		Sí
PREGUNTA 4	¿Cómo está la salud de la persona mayor?	Ningún síntoma/todos bien
		Falta de aire
		Tos
		Cansancio
		Dolores
		Fiebre
		Congestión nasal
		Sin olfato y/o gusto
PREGUNTA 4.1	¿Cuánto tiempo?	Número en días
PREGUNTA 5	¿La persona mayor se hizo la prueba rápida de COVID?	No
		Sí
PREGUNTA 5.1	Si es así, ¿cuál fue el resultado?	Negativo
		Positivo
PREGUNTA 5.2	Si es positivo, ¿cuál fue el resultado?	No se aplica
		IGM* positivo
		IGG^†^ positivo
PREGUNTA 6	¿El cuidador está aislado?	No
		Sí
		No se aplica
PREGUNTA 7	¿Cómo está la salud del cuidador?	Ningún síntoma/todos bien
		Falta de aire
		Tos
		Cansancio
		Dolores
		Fiebre
		Congestión nasal
		Sin olfato y/o gusto
		No se aplica
PREGUNTA 7.1	¿Cuánto tiempo?	Número en días
PREGUNTA 8	¿El cuidador se hizo la prueba rápida de COVID?	No
		Sí
		No se aplica
PREGUNTA 8.1	Si es así, ¿cuál fue el resultado?	Negativo
		Positivo
		No se aplica
PREGUNTA 8.2	Si es positivo, ¿cuál fue el resultado?	No se aplica
		IGM* positivo
		IGG^†^ positivo
PREGUNTA 9	¿Cómo está la salud de los miembros de la familia?	Ningún síntoma/todos bien
		Falta de aire
		Tos
		Cansancio
		Dolores
		Fiebre
		Congestión nasal
		Sin olfato y/o gusto
		No se aplica
PREGUNTA 9.1	¿Cuánto tiempo?	Número en días
PREGUNTA 10	¿Alguien más en la casa se hizo la prueba rápida?	No
		Sí
PREGUNTA 10.1	Si es así, ¿quién? *Considerar personas además de la persona mayor y el cuidador. Ejemplo: si el cuidador es el cónyuge, esta pregunta no debe ser considerada, pero si el cuidador es el hijo y el esposo se hizo la prueba, esta pregunta debe ser respondida*	No se aplica
		Cónyuge de la persona mayor
		Hijo(a) de la persona mayor
		Nieto(a) de la persona mayor
		Padres de la persona mayor
		Hermano(a) de la persona mayor
		Cónyuge del cuidador
PREGUNTA 10.2	Si es así, ¿cuál fue el resultado?	No se hizo la prueba
		Negativo
		Positivo
PREGUNTA 10.3	Si es positivo, ¿cuál fue el resultado?	No se aplica
		IGM* positivo
		IGG^†^ positivo
INTERVENCIÓN 10		Cuando tenga síntomas leves, orientar los cuidados en casa - VER flujo de atención COVID-19
PREGUNTA 11	¿Quién hace las compras?	No salen de casa/piden por teléfono/piden por aplicación
		La propia persona mayor
		El cuidador
		Cónyuge de la persona mayor
		Hijo(a) de la persona mayor
		Nieto(a) de la persona mayor
		Padres de la persona mayor
		Hermano(a) de la persona mayor
		Sobrino(a) de la persona mayor
		Yerno de la persona mayor
		Nuera de la persona mayor
		Vecino(a) de la persona mayor
PREGUNTA 12	¿Alguien en la casa trabaja? ¿Y sale de casa para trabajar?	Nadie trabaja
		Trabaja en Actividades Esenciales y sale de casa
		Trabaja en Actividades No Esenciales y sale de casa
		Trabaja en Actividades No Esenciales y está en *home office*
PREGUNTA 13	¿Alguien ha viajado en los últimos 14 días?	No
		Sí
PREGUNTA 14	¿Contactantes en los últimos 14 días?	No
		Sí
PREGUNTA 15	¿La persona mayor ya fue vacunada contra la gripe?	No
		Sí
PREGUNTA 15.1	¿Quién la vacunó?	No se aplica
		SAD^‡^
		USF^§^
PREGUNTA 16	¿El cuidador ya fue vacunado contra la gripe?	No
		Sí
		No se aplica
PREGUNTA 16.1	¿Quién la vacunó?	No se aplica/No se vacunó
		SAD^‡^
		USF^§^
INTERVENCIÓN 11		Explicar la diferencia entre gripe y coronavirus y la protección que ofrecen las vacunas.
PREGUNTA 17	¿Hubo cambio en la rutina para adaptarse al cuidado de la persona mayor/su cuidado?	No
		Sí
INTERVENCIÓN 12		Enseñar al cuidador estrategias de mantenimiento de los cuidados de salud para disminuir la contaminación (higiene y antisépticos).
INTERVENCIÓN 13		Enseñar a mantener la propia salud física y mental (sueño, ejercicios en casa, música relajante, alimentación).
INTERVENCIÓN 14		Determinar las necesidades de mejoras en casa para compensar la higiene y la salud mental, detallar.
INTERVENCIÓN 15		Determinar las necesidades de cambios relacionados con la seguridad en casa, detallar.
INTERVENCIÓN 16		Orientar sobre condiciones adecuadas para la vivienda con la persona mayor, detallar.
INTERVENCIÓN 17		Cuando tosa o estornude, cubra la boca y la nariz con un pañuelo desechable y tírelo a la basura, o cúbrase con el antebrazo. No use sus manos para evitar transmitir el virus a objetos y personas.
INTERVENCIÓN 18		Lave sus manos con agua y jabón durante al menos 20 segundos con frecuencia. Cuando no sea posible, límpiese y desinféctese las manos con alcohol en gel al 70%.
INTERVENCIÓN 19		Evite el contacto cercano con personas que tengan síntomas de resfriado o gripe.
INTERVENCIÓN 20		Evite el contacto cercano con personas mayores, embarazadas, mujeres en periodo de lactancia o personas que se enferman con facilidad.
INTERVENCIÓN 21		No comparta objetos de uso personal, como vasos, cubiertos y platos, y limpie objetos que se toquen con frecuencia, como celulares.
PREGUNTA 18	¿De los cuidados que realizan con la persona mayor, hay alguno que presenta dificultad?	Ninguno
		Ducharse
		Higiene personal: usar el baño, limpiarse o arreglarse la ropa
		Vestirse
		Alimentarse
		Transferencia: Levantarse y caminar
		Vendaje
INTERVENCIÓN 22		Fomentar la independencia, pero ayudar a la persona mayor cuando ella no pueda hacerlo.
INTERVENCIÓN 23		Determinar la capacidad mental y cognitiva de la persona mayor para asumir las actividades de autocuidado.
INTERVENCIÓN 24		Orientar al cuidador para que anime a la persona mayor a asumir la mayor responsabilidad posible por su propio autocuidado.
INTERVENCIÓN 25		Apoyar al cuidador en el establecimiento de límites y en el cuidado de sí mismo.
INTERVENCIÓN 26		Enseñar al cuidador estrategias de mantenimiento de los cuidados de salud para mantener la propia salud física y mental.
INTERVENCIÓN 27		Fomentar la verbalización de sentimientos, percepciones y miedos en relación con el baño.
INTERVENCIÓN 28		Orientar al cuidador para que establezca una relación de confianza con la persona mayor durante el baño.
INTERVENCIÓN 29		Orientar al cuidador para que anime a la persona mayor a verbalizar señales y síntomas relacionados con dolor, sensibilidad, picazón, alteración en la audición, zumbido, vértigo, alteración en la fuerza muscular, integridad de la piel y edemas.
INTERVENCIÓN 30		Orientar al cuidador para lavar y acondicionar el cabello de la persona mayor, masajeando con la punta de los dedos el cuero cabelludo, y preferir el uso de jabón líquido y neutro durante el baño.
INTERVENCIÓN 31		Orientar al cuidador para realizar la limpieza de las uñas, aplicar cremas en la piel, especialmente en las prominencias óseas, peinar el cabello con peines de dientes anchos, secar cuidadosamente los pliegues corporales y entre los dedos, y mantener el perineo seco y limpio en intervalos regulares.
INTERVENCIÓN 32		Orientar al cuidador sobre los signos y síntomas de retención urinaria/infección urinaria, que son disminución de la frecuencia, consistencia, olor, volumen, color, dolor y globo vesical, según corresponda.
INTERVENCIÓN 33		Orientar sobre la ocurrencia de signos y síntomas de diarrea, estreñimiento, impactación e incontinencia fecal.
INTERVENCIÓN 34		Orientar al cuidador para que, ante cualquier episodio vesico-intestinal, se realice una higiene adecuada (agua y jabón).
INTERVENCIÓN 35		Orientar al cuidador para disponer la ropa necesaria para la persona mayor en un área accesible.
INTERVENCIÓN 36		Orientar al cuidador para ayudar a la persona mayor en el uso de cordones, botones y cremalleras.
INTERVENCIÓN 37		Orientar al cuidador a estimular a la persona mayor a vestirse sola de acuerdo con su condición física.
INTERVENCIÓN 38		Orientar al cuidador a estimular la verbalización de la persona mayor sobre sus limitaciones para vestirse.
INTERVENCIÓN 39		Orientar al cuidador a monitorear la condición de la boca de la persona mayor: labios, lengua, membranas mucosas, dientes, encías y aparatos dentales y su adaptación. Atención a signos de inflamación, encías esponjosas, retraídas o con sangrado; labios secos, agrietados; heridas; lengua cruda, escarlata; e hiperemia e hipertrofia de las papilas.
INTERVENCIÓN 40		Orientar al cuidador a posicionar correctamente a la persona mayor para facilitar la masticación y la deglución, proporcionando apoyo adecuado para el cuello.
INTERVENCIÓN 41		Orientar al cuidador a ofrecer la comida de forma apetitosa, a la temperatura adecuada, arreglada en el plato, si es necesario, cortando la carne, pelando los huevos, abriendo los paquetes y/o alimentos empaquetados.
INTERVENCIÓN 42		Orientar al cuidador a ofrecer cubiertos y dispositivos según la necesidad de la persona mayor, como pajillas y cucharas.
INTERVENCIÓN 43		Orientar al cuidador a ayudar y/o incentivar a la persona mayor a mantener la rutina de higiene bucal.
INTERVENCIÓN 44		Orientar al cuidador a observar señales no verbales de incomodidad, como expresiones faciales y movimientos excesivos en personas mayores que son incapaces de comunicarse efectivamente.
INTERVENCIÓN 45		Orientar al cuidador o a la familia a obtener herramientas para ayudar en las actividades diarias: andador, bastón, muletas, barras de apoyo, iluminación adecuada, esquinas redondeadas y barandillas si es necesario.
INTERVENCIÓN 46		Orientar al cuidador o a la familia a controlar factores ambientales que puedan influir de manera negativa en la movilidad como la temperatura del ambiente, calor o frío, iluminación y ruidos.
INTERVENCIÓN 47		Orientar al cuidador a reducir factores que precipiten o aumenten la experiencia del dolor como miedo, fatiga, monotonía y falta de información.
INTERVENCIÓN 48		Orientar sobre el uso adecuado de analgésicos prescritos para ayudar con el dolor cuando esté relacionado con el movimiento.
INTERVENCIÓN 49		Orientar sobre el cambio de posición cada 2 horas, protegiendo prominencias óseas y evitando edema.
INTERVENCIÓN 50		Orientar al cuidador a animar a la persona mayor a involucrarse en los cambios de posición cuando sea posible.
INTERVENCIÓN 51		Orientar al cuidador a mantener la cama de la persona mayor limpia y con sábanas estiradas para evitar tensiones en heridas y deslizamientos durante la movilización.
INTERVENCIÓN 52		Orientar al cuidador a utilizar equipos apropiados para apoyar miembros y prominencias óseas, como cojines.
PREGUNTA 19	¿La persona mayor desarrolló algún comportamiento diferente durante este distanciamiento social?	Ninguna/no
		Agitación
		Tristeza
		Agresividad
		Confusión
		Trastornos de memoria
		Trastornos de orientación
		Trastornos de comportamiento
		Ansiedad
		Miedo
INTERVENCIÓN 53		Orientar al paciente en el tiempo, espacio y persona.
INTERVENCIÓN 54		Proporcionar un calendario.
INTERVENCIÓN 55		Estimular la cognición mediante ejercicios como pintura, dibujo, rompecabezas, cantar y escuchar música, usar televisión y radio, colocar fotos de familiares cercanos al paciente.
INTERVENCIÓN 56		Orientar sobre el ambiente en el que la persona mayor duerme, oscurecer el ambiente cuando sea la hora de dormir, tener una ventilación adecuada.
INTERVENCIÓN 57		Utilizar técnicas calmantes como música de preferencia de la persona mayor, tocarla y estar presente.
INTERVENCIÓN 58		Reconocer los miedos y los sentimientos del paciente.
INTERVENCIÓN 59		Permitir que la persona mayor mantenga algunos de sus rituales para limitar la ansiedad.
INTERVENCIÓN 60		Informar la percepción del cuidador de manera calmada, tranquilizadora y no argumentativa.
INTERVENCIÓN 61		Responder al tono, en lugar del contenido, de la alucinación o delirio.
INTERVENCIÓN 62		Mantener un ambiente bien iluminado que reduzca contrastes agudos y sombras.
PREGUNTA 20	¿Hubo uso de servicios de salud?	No
		Sí
DESENLACE		Continuó con el cuidado del SAD^‡^ después del término de las llamadas.
		No quiso seguir recibiendo la visita presencial del SAD^‡^ por causa de la pandemia ni las llamadas de la investigación (firmó el término y no retiró la participación en la investigación).
		Alta del SAD^‡^ después de las llamadas o término de las llamadas
		Internación
		Fallecimiento
Motivo de la internación o fallecimiento		Descriptivo

*IGM = Imunoglobulina M; ^†^IGG = Imunoglobulina G; ^‡^SAD = Servicio de Atención Domiciliaria; ^§^USF = Unidad de Salud de la Familia

El instrumento de la Figura 2 fue desarrollado a la luz de la evaluación gerontológica y fundamentado en la Teoría de la Transición^12^ ^),(^ ^19^. Se elaboró de forma sistematizada con el objetivo de desarrollar un plan adecuado de cuidados y apoyo en la intervención del estado general de la persona mayor y de su cuidador relacionado al cuidado de ambos durante la investigación del síndrome gripal y aislamiento social en tiempos de COVID-19.

Las preguntas y las intervenciones del telecuidado fueron elaboradas de acuerdo con las atribuciones legales del profesional enfermero, basadas en los diagnósticos de riesgo de tensión del papel del cuidador, riesgo de infección y riesgo de síndrome del anciano frágil de la Taxonomía de NANDA-I^14^, asociados estos diagnósticos, respectivamente, a las intervenciones del NIC^15^, partieron de las clasificaciones de mejora del enfrentamiento; apoyo al cuidador; control de infección y protección contra infección; asistencia del autocuidado, de las actividades esenciales de la vida diaria, baño/higiene, vestir/arreglarse, alimentación y transferencia; estimulación cognitiva y facilitación de la autorresponsabilidad y en el Protocolo de Manejo Clínico del Coronavirus (COVID-19) en la Atención Primaria a la Salud del Ministerio de Salud^20^. En cuanto a la comparación de las evaluaciones e intervenciones en la salud de la persona mayor y de su cuidador mediante la teleconsulta, se utilizó los dominios de NANDA I y NIC.

Estos diagnósticos e intervenciones fueron elegidos para la promoción y prevención de la salud de la persona mayor y de sus cuidados en el aislamiento social y con el objetivo de controlar la infección por COVID-19.

Cuando se identificaba alguna situación que demandaba urgencia/emergencia o atención presencial, en el momento de la llamada, el equipo del SAD era activado por el equipo de investigación y realizaba la debida atención o seguimiento.

El instrumento fue validado por cinco especialistas en gerontología en marzo de 2020 y se aplicó a siete personas mayores registradas en el servicio de atención domiciliaria que no fueron incluidas en el estudio antes del inicio de la recolección de datos. Posteriormente, se publicaron datos preliminares que indicaron la validación del instrumento^21^.

### Tratamiento y análisis de datos

Los datos se tabularon utilizando el *software Microsoft Exce*l 2016. Posteriormente, fueron importados al *software* estadístico IBM-SPSS (*Statistical Package for the Social Sciences*) versión 24, para el correspondiente tratamiento analítico. Se utilizaron estadísticas descriptivas dispuestas en forma de tabla, donde se contabilizaron las frecuencias y porcentajes de las variables en estudio. Tras evaluaciones iniciales de los datos, se aplicó métodos estadísticos para verificar asociaciones y correlaciones entre las variables. Para evaluar el comportamiento entre dos variables categóricas se utilizó el test estadístico de Fisher (Test de Asociación), y, para la evaluación de diferencias entre las medias, se aplicó el test de Kruskal Wallis. 

### Aspectos éticos

La investigación fue aprobada por el Comité de Ética en Investigación con Seres Humanos de la institución sede de la investigación, con los números de Certificado de Presentación de Apreciación Ética (CAAE) 14354919.1.0000.5243 y 43682821.6.0000.5243. Se celebró un acuerdo de cooperación técnica con el municipio al que estaba vinculado el SAD. En cuanto al reclutamiento de participantes, el equipo de investigación realizó el primer contacto telefónico para explicar la investigación que sería realizada, obteniendo inicialmente el consentimiento verbal de participación mediante la lectura del Consentimiento Informado (IC) tanto para la persona mayor como para el cuidador. Se resaltó el sigilo de la información. Posteriormente, durante la visita presencial por parte del equipo del SAD en colaboración con el equipo de investigación, se obtuvo la firma del Consentimiento Informado de todos los participantes.

No se observaron riesgos directos a los participantes, ya que las intervenciones de enfermería ofrecidas a través de las llamadas telefónicas ya formaban parte del espectro de orientaciones y cuidados de enfermería previamente ofrecidos a la población del SAD. Se observó un leve malestar que pudo haber sido causado por los inconvenientes relacionados con las llamadas y las posibles alteraciones emocionales en referencia a las preguntas e intervenciones del cuestionario, que fueron minimizados por los investigadores mediante la escucha activa y la oferta de soporte psicológico o médico del equipo multiprofesional del SAD. El contacto telefónico de los investigadores estuvo disponible para casos de intercurrencias, orientaciones y encaminamientos. Se ofrecieron cuidados de enfermería relativos a la promoción de la salud por medio de orientaciones sobre higiene, aglomeración, respeto al aislamiento social y mantenimiento de mejores condiciones de vida en la pandemia de COVID-19, con mejora de la capacidad emocional y funcional.

Los beneficios del estudio abordan la contribución social, académica y profesional para la consolidación de un cuidado de enfermería innovador en el órgano público, de bajo costo y fácil manejo, utilizado en el apoyo al servicio de los equipos de enfermería, promoviendo ayuda en la promoción de la salud, adherencia al tratamiento, confort psicológico, rastreo y prevención de complicaciones de salud de la persona mayor.

Tras la finalización del estudio, el grupo de control recibió todas las llamadas con las debidas intervenciones de enfermería en el mismo período.

## Resultados

El presente estudio contó con un total de 1691 llamadas, de estas, 1515 para los 131 participantes del grupo intervención y 176 para los 88 participantes del grupo control, mediante el uso de los instrumentos de llamada adecuados para cada grupo.

Los resultados de la Tabla 1 detallan las características de los participantes del estudio, así como la separación por grupo analizado (control e intervención). El Test Exacto de Fisher se aplicó para evaluar la distribución porcentual de la característica en relación con los grupos analizados, es decir, su homogeneidad.

**Tabla 1:** Evaluación de la homogeneidad de la muestra de personas mayores y cuidadores. São Gonçalo, RJ, Brasil, 2020

Característica	Grupo				Total		P-valor*
	Control		Intervención				
	n	%	n	%	N	%	
**Sexo de la persona mayor**							0,165
Masculino	44	50,0	52	39,7	96	43,8	
Femenino	44	50,0	79	60,3	123	56,2	>0,999
**Sexo del cuidador**							
Masculino	13	15,7	17	15,2	30	15,4	
Femenino	70	84,3	95	84,8	165	84,6	
**Edad (promedio)**							
Persona mayor	75,2		75,9				0,849
Cuidador	51,6		53,1				0,615
**Posee cuidador**							
Sí	83	94,3	112	85,5	195	89,0	0,048
No	5	5,7	19	14,5	24	11,0	0,068
**Número de comorbilidad en las personas mayores**							
0	0	0,0	4	3,1	4	1,8	
1	7	8,0	14	10,7	21	9,6	
2	21	23,9	37	28,2	58	26,5	
3	22	25,0	29	22,1	51	23,3	
4	13	14,8	28	21,4	41	18,7	
5	11	12,5	14	10,7	25	11,4	
6	6	6,8	4	3,1	10	4,6	
7	4	4,5	1	0,8	5	2,3	
8	3	3,4	0	0,0	3	1,4	
9	1	1,1	0	0,0	1	0,5	0,709
**Número de comorbilidad en los cuidadores**							
0	44	50,0	70	53,4	114	52,1	
1	28	31,8	34	26,0	62	28,3	
2	15	17,0	23	17,6	38	17,4	
3	1	1,1	4	3,1	5	2,3	

*Test Exacto de Fisher, significativa se p-valor < 0,05

Entre las características analizadas en cuanto a la homogeneidad, la única que mostró significancia fue “Posee cuidador”, ya que se observaron las diferencias porcentuales entre los grupos (p-valor = 0,048), indicando que el grupo control tuvo un mayor porcentaje de personas mayores con cuidador (94,3%) en comparación con el de intervención (85,5%).

Se realizó una evaluación de la homogeneidad de las variables cuantitativas respecto a: edad, edad del cuidador, número de comorbilidades de las personas mayores y número de comorbilidades de los cuidadores, por medio de los resúmenes estadísticos y aplicación del test de Kruskal-Wallis, con el objetivo de evaluar si la distribución de los valores entre el grupo control e intervención eran similares. Se destaca que el test de Kruskal-Wallis es significativo si el p-valor es < 0,05.

En este sentido, se observó que solo la comparación ‘Número de comorbilidades en las personas mayores’ en el grupo de control fue mayor que en el grupo de intervención (p-valor = 0,023). Las demás comparaciones no fueron significativas.

En relación con las evaluaciones de la salud de la persona mayor y de los cuidadores respecto a los signos y síntomas de COVID-19, como: falta de aire, tos, cansancio, dolores, fiebre, coriza y la ausencia de gusto y/o olfato, durante las llamadas, también se aplicó el test de Kruskal-Wallis con significancia si el p-valor es < 0,05. En esta evaluación, no hubo diferencias porcentuales entre el grupo control y el intervención.

En cuanto a la comparación de las evaluaciones de la salud del cuidador en relación a los grupos analizados, se observó significancia en la opción “Ningún síntoma” favoreciendo al grupo control (p-valor < 0,001). Este último tuvo más evaluaciones positivas que el grupo intervención.

En la comparación de las evaluaciones de la salud de los residentes en relación a los grupos analizados, el resultado más positivo fue “sin síntomas” en el grupo intervención con (p-valor < 0,001).

En la Tabla 2, se puede observar el número medio de intervenciones realizadas con la persona mayor y con el cuidador durante las llamadas en el grupo intervención.

**Tabla 2:** Número medio de intervenciones realizadas en el grupo intervención con las personas mayores y cuidadores. São Gonçalo, RJ, Brasil, 2020

Semana de llamada	Dominio 7 - Roles y Relacionamiento				Dominio 11 - Seguridad/protección				Dominio 1 - Promoción de la salud			
	Diagnóstico - Riesgo de Tensión del Rol del Cuidador				Diagnóstico - Riesgo de Contaminación				Diagnóstico - Riesgo de Síndrome del Anciano Frágil			
	Promedio	IC* de 95%		P-valor^§^	Promedio	IC* de 95%		P-valor^§^	Promedio	IC* de 95%		P-valor^§^
		Inferior^†^	Superior^‡^			Inferior^†^	Superior^‡^			Inferior^†^	Superior^‡^	
1	4,73	4,39	5,06	**<0,001**	8,97	8,67	9,27	**<0,001**	4,99	4,02	5,96	**<0,001**
2	2,07	1,82	2,32		3,19	2,67	3,72		0,26	0,1	0,42	
3	1,85	1,62	2,08		2,41	1,93	2,88		0,15	0,04	0,27	
4	1,68	1,45	1,91		2,02	1,62	2,42		0,14	0,02	0,26	
5	1,6	1,4	1,81		1,5	1,25	1,76		0,1	0	0,2	
6	1,5	1,32	1,68		1,57	1,25	1,89		0,13	0	0,25	
7	1,47	1,3	1,65		1,62	1,28	1,95		0,04	0	0,11	
8	1,45	1,29	1,61		1,58	1,23	1,94		0,07	0	0,16	
9	1,45	1,29	1,62		1,57	1,21	1,93		0,02	0	0,06	
10	1,48	1,3	1,66		1,73	1,34	2,13		0,01	0	0,03	
11	1,49	1,31	1,67		1,71	1,29	2,13		0,03	0	0,09	
12	1,56	1,34	1,78		1,83	1,38	2,27		0,03	0	0,09	
13	1,69	1,38	1,99		1,82	1,38	2,26		0,02	0	0,06	
14	1,63	1,36	1,89		1,81	1,34	2,27		0,02	0	0,07	

*IC = Intervalo de confianza; ^†^Inferior = Estadística; ^‡^Superior = Estadística; ^§^P-valor = Prueba Exacta de Fisher, significativa si p-valor < 0,05

Se observa que en la primera llamada hay una mayor necesidad de intervenciones. A lo largo de las llamadas, la cantidad disminuye y este resultado fue significativo (p-valor < 0,001) para todos los aspectos estudiados, pero principalmente para la promoción de la salud, que trata de la fragilidad y se estabiliza con el tiempo, mientras que la Seguridad/protección permaneció como algo necesario y fue reforzado con la pandemia de COVID-19, asociada a la promoción del bienestar del cuidador incluido en el dominio Roles y Relación.

En la Tabla 3, se evalúa la existencia de diferencia en el porcentaje de respuestas de cada desenlace en relación al grupo control e intervención. Hay diferencias en los desenlaces analizados (p-valor < 0,001). Se observa que en el desenlace de alta, el resultado fue porcentualmente significativo en el grupo intervención. Entonces, de los casos que tuvieron alta del SAD (36), casi el 84% fueron en el grupo de intervención. Es importante destacar que la alta del SAD fue realizada por el equipo del servicio conforme avanzaba la investigación y las evaluaciones del paciente fueron positivas.

**Tabla 3:** Comparación entre desenlace del grupo control e intervención de personas mayores y cuidadores. São Gonçalo, RJ, Brasil, 2020

Desenlace	Grupo				Total		P-valor*
	Control		Intervención				
	n	%	n	%	n	%	
1 Continuó con el cuidado del SAD^†^ después del término de las llamadas	67	44,4	84	55,6	151	100	**<0,001**
2 Alta del SAD^†^ después de las llamadas o término de las llamadas	6	16,7	30	83,3	36	100	
3 Internación	11	64,7	6	35,3	17	100	
4 Fallecimiento	4	26,7	11	73,3	15	100	

*P-valor = Test Exacto de Fisher, significativa se p-valor < 0,05; ^†^SAD = Servicio de Atención Domiciliaria

Para ponderar estos resultados de la mejor manera, se aplicó el uso de la Razón de Chances y los resultados se compararon entre el desenlace “alta” con los demás desenlaces. En este análisis, se observa que la probabilidad de resultado de alta en el grupo de intervención fue mayor en comparación con otros desenlaces en el grupo de control. Así, tener un resultado de “alta” tiene casi nueve veces más probabilidades de ocurrir en el grupo de intervención que en el grupo de control.

## Discusión

Ante el momento de transición en la salud experimentado en el mundo debido a la pandemia de COVID-19, el consecuente cambio en los modos de vida del binomio persona mayor y sus cuidadores atendidos por el SAD ha proporcionado la posibilidad de utilizar los recursos tecnológicos mediados por la teleenfermería, en el apoyo a la continuidad del cuidado de las personas mayores y sus cuidadores asistidos por el servicio.

De forma inédita, el estudio se desarrolló en el auge de la pandemia de COVID-19, basado en la cientificidad de la combinación de la Teoría de la Transición^12^ ^),(^ ^19^, del Sistema de Lenguaje Estandarizado NANDA I^14^ y NIC^15^ para la orientación documental del instrumento de llamada y también la reglamentación de la Resolución COFEN 696/2022^22^. La combinación de la teoría de transición a través de los patrones de respuestas permitió fundamentar la recolección de datos de los indicadores del proceso de cuidado de la persona mayor y de sus cuidadores, los cuales son: sentirse conectados con los profesionales, interactuar aunque sea por teléfono, estar situados y desarrollar confianza y *coping*.

Las lenguas estandarizadas de las Taxonomías de NANDA I^14^ y NIC^15^ fueron cruciales en la identificación de los principales diagnósticos de Enfermería como: Síndrome del Anciano Frágil, Riesgo de Tensión del rol del cuidador y Riesgo de Contaminación. La Taxonomía de NIC consistió en las principales intervenciones mediante los respectivos diagnósticos.

Estudios internacionales en la década de 1990 ya señalaban que aproximadamente la mitad de todos los pacientes que van a los cuidados domiciliarios necesitan continuidad de cuidados de enfermería calificados. En dichos estudios, se destaca que, en el momento del alta hospitalaria, los pacientes aún están muy debilitados y no recuperados lo suficiente como para aprender los procedimientos necesarios de autocuidado^23^.

Con esto, surge el ambiente de atención domiciliaria a través de la Tecnología *TeleHomeCare*; es decir, los estudios también preveían que esta tecnología ayudaría a proporcionar el nivel necesario de atención y servicios para ejercer los cuidados domiciliarios a las personas mayores y sus cuidadores por las enfermeras registradas en atención domiciliaria y competentes en *TeleHomeCare*. Ante esto, los estudios ya demostraban que las situaciones de calamidad pública, la creatividad y la búsqueda de estrategias de atención remota superan los desafíos impuestos por las medidas de distanciamiento y pasan a ocupar la agenda de los sistemas de salud en los diferentes contextos de cuidado^23^.

Al considerar el estado de calamidad pública y la propuesta del estudio, fue posible unir el equipo de investigación y el equipo multiprofesional del SAD a través de entrenamientos, asociando el uso de la tecnología y poner en práctica la planificación de cuidados de enfermería contenida en las cinco etapas ininterrumpidas del Proceso de Enfermería sistematizado y compuesto en el instrumento de llamada. La teleenfermería permitió proporcionar una asistencia de apoyo y de continuidad innovadoras a las personas mayores y a sus cuidadores que estaban internados en el domicilio en ese momento de aislamiento social y que no tenían acceso a los servicios específicos de salud.

Cabe resaltar que los profesionales de salud necesitaron ser capacitados para desarrollar habilidades/competencias innovadoras para garantizar la calidad, la seguridad y la eficiencia del cuidado mediante la tecnología. Esto que justifica la creación del instrumento de llamadas basado en la transición del cuidado presencial al remoto durante la pandemia de COVID-19^24^.

Así como las demás reglamentaciones de acceso a los datos de la sociedad, la teleenfermería también debe ser previamente consentida por el paciente o por su representante legal, y su realización es responsabilidad del enfermero. El registro de la teleconsulta fue obligatorio y basado en el proceso de enfermería (consulta de enfermería, diagnóstico, planificación, intervención y evaluación), para la organización y orientación del cuidado de las personas mayores y sus cuidadores. De este modo, en las llamadas, las personas mayores y sus cuidadores del SAD fueron abordados en cuanto a la recolección de datos basada en la combinación de la teoría de transición, los diagnósticos fueron extraídos de los dominios de NANDA-I^14^: roles y relaciones familiares, seguridad/protección y promoción de la salud.

En el Dominio I Promoción de la Salud, se extrajo el diagnóstico Riesgo de Síndrome del Anciano Frágil, ya que, a pesar de no haber una definición consensuada, la fragilidad puede entenderse como un síndrome multidimensional que implica la interacción compleja de factores biológicos, psicológicos y sociales. Esa interacción culmina en una mayor vulnerabilidad y está asociada a los riesgos de desenlaces clínicos como el declive funcional, caídas, desnutrición, hospitalización y muerte. Por lo tanto, un plan de cuidados de enfermería en cuanto a estos signos y síntomas fue esencial. Las intervenciones de NIC se relacionaron con el diagnóstico de enfermería en cuanto a la prevención de complicaciones agudas, a la orientación para la adopción de una alimentación equilibrada, cuidados personales y para mantenerse activo^25^.

En el Dominio 7 Roles y Relaciones, la justificación también se dio mediante la mejor cobertura asistencial a la persona mayor y sus cuidadores durante la pandemia de COVID-19. En este dominio, se debió a la posible sobrecarga física y emocional del cuidador de la persona mayor. Las intervenciones de NIC se justificaron con el objetivo de minimizar los efectos perjudiciales de la tensión del rol del cuidador a través de las orientaciones de enfermería dispuestas en el instrumento de llamada. En el dominio 11 Seguridad y Protección, se destacó el Riesgo de Infección durante la pandemia de COVID-19. Las intervenciones también se guiaron de forma sistematizada mediante el instrumento de llamada.

Durante las llamadas, se identificó y confirmó que las personas mayores y sus cuidadores poseían enfermedades crónicas y eran acompañados por el SAD y la Red de Asistencia a la Salud del municipio sin ninguna regularidad. Estudios corroboran que con el agravamiento de la pandemia de COVID-19, el esfuerzo del cuidado, en general, aumentó significativamente y esto se reflejó en la salud física y mental de la población, en especial de las mujeres en que, casi una de cada dos, tiene comorbilidades crónicas que se agravaron durante la pandemia. Consecutivamente, los sentimientos de aislamiento, tristeza y ansiedad por parte de este binomio - personas mayores y sus cuidadores - también aumentaron^26^ ^)-(^ ^27^.

Este dato coincide con el presente estudio, considerando el alto porcentaje de personas mayores que tenían cuidadoras del género femenino, siendo esa recurrencia mayor en comparación al género masculino, como se pudo ver en la Tabla 1. También se identificó el género femenino para cuidadoras que no recibían remuneración, lo cual coincide con estudios sobre el cuidado prestado a las personas mayores^28^.

En las llamadas realizadas a la persona mayor y sus cuidadores, fue posible observar la necesidad de una mirada diferenciada del Estado, la sociedad y las familias en cuanto a la organización y planificación familiar. Estudios ya narran la importancia de incluir al cuidador no solo como ejecutor de las acciones planificadas, sino como sujeto principal en la promoción y calidad del cuidado, cuando la persona mayor pierde la capacidad de autocuidado.

La visibilidad del rol del cuidador fue relevante en el estudio ya que, a través de él, fue posible llevar asistencia de salud al Binomio persona mayor y cuidador. Políticas públicas de salud son necesarias para la cobertura del cuidado integral a la salud de la persona mayor y sus cuidadores, ya que el cuidador está en la “primera línea anónima” del cuidado a la persona mayor, no solo durante la pandemia de COVID-19, sino como requisito para la adhesión de la persona mayor al Servicio de Atención Domiciliaria^29^.

La teleenfermería reveló un número significativo de intervenciones en la primera llamada y se mantuvo durante las primeras cuatro semanas debido al pánico que se instaló en los ciudadanos, las incertidumbres y los fallecimientos provocados por la pandemia. El estado pandémico no permitía las visitas presenciales del equipo del SAD y la asistencia a la salud de la población en general se vio perjudicada.

En este sentido, la teleenfermería como apoyo en las intervenciones de enfermería en la continuidad del cuidado a la persona mayor y sus cuidadores fue innovadora y esencial para resolver las dudas de los pacientes y reducir el miedo a la interacción que, de acuerdo con las noticias transmitidas en los medios, era predictoras de fallecimientos. En este episodio, el equipo de llamadas pudo desarrollar sus acciones de enfermería con maestría y adquirir la confianza de la persona mayor y sus cuidadores asistidos por el SAD.

Después de las cuatro semanas, se pudo observar que la cantidad de llamadas disminuyó en el aspecto del diagnóstico del Riesgo de Síndrome del Anciano Frágil y se equiparó a las demás intervenciones de Riesgo de Contaminación y Riesgo de Tensión del Rol del Cuidador, resultando en un resultado positivo (p-valor < 0,001).

Las recomendaciones de seguridad para las personas mayores fueron mucho más allá de las que ya se realizaban diariamente por el cuidador. Fue necesario intensificar los cuidados y la atención para acciones preventivas vinculadas directamente al comportamiento y la higiene, ya que el momento era de mucha soledad y dudas respecto a la enfermedad^19^ ^),(^ ^22^ ^),(^ ^30^ ^)-(^ ^31^.

Otros resultados se asemejan a estudios nacionales e internacionales, sobre todo, en lo que respecta a la continuidad de las personas mayores y sus cuidadores en el SAD, recibir alta, ser internados o fallecer después de las llamadas o al término de las llamadas. Hubo un destaque de 83,3% para el grupo intervención, ya que en el auge de la COVID-19, las internaciones se destinaban a los casos de mayor gravedad como el síndrome de insuficiencia respiratoria aguda, lo que culminó en el aumento de altas de los pacientes de toda la Red de Asistencia a la Salud y también en los adheridos al SAD^26^ ^)-(^ ^27^. Es importante resaltar que la alta de la persona mayor y sus cuidadores asistidos por el SAD, durante la investigación fue posible gracias a la información actualizada que el equipo de investigación transmitía al equipo multiprofesional del SAD, siendo que esta última también prestaba la continuidad del cuidado a través de la tecnología hasta la posible alta.

Dado los resultados significativos, fue notoria la importancia de los profesionales de salud en la minimización de los efectos causados por la calamidad pública de salud durante la pandemia de COVID-19, especialmente en relación con los trabajadores de enfermería, protagonistas en el control de la transmisión de esta enfermedad, tanto en la primera línea como en las intervenciones de apoyo a la continuidad del cuidado de la persona mayor y sus cuidadores por intermedio de la teleenfermería.

Por lo tanto, los resultados mostraron que la teleenfermería realizada por el enfermero como tecnología de apoyo en la transición del cuidado a las personas mayores y sus cuidadores durante la pandemia de COVID-19 obtuvo un índice de confianza del 95% y significancia (p-valor < 0,001) en todos los dominios y diagnósticos estudiados (Roles y Relaciones - Riesgo de Tensión del Rol del Cuidador; Seguridad y Protección - Riesgo de Contaminación y Promoción de la Salud - Riesgo de Síndrome del Anciano Frágil). Se destaca la importancia de la teleenfermería en las intervenciones realizadas por el equipo de enfermeros, con resultados positivos en la promoción de la salud de la persona mayor y sus cuidadores.

En términos de información, la salvaguarda de las informaciones y de los registros obtenidos durante la teleenfermería es responsabilidad del profesional que la ejecutó y/o de la institución de salud vinculada. La práctica de la teleenfermería requiere adecuaciones de las partes involucradas - enfermero y paciente - que van más allá de la infraestructura y conectividad^21^. En este sentido, el enfermero dirige la información al equipo del SAD para certificar el fallecimiento.

El “hacer salud” es realizado por el equipo de enfermería o equipo multiprofesional en los más diversos contextos, de forma que cada categoría profesional posee su marco de saberes y prácticas que objetivan el diagnóstico, la prevención y la recuperación de enfermedades y agravios que afectan al individuo, la familia y la colectividad. Es importante saber que la teleenfermería debe ser desarrollada por un profesional de enfermería con competencia técnica y autonomía y que sepa distinguir una práctica avanzada de enfermería a la de una generalista^32^.

El soporte de la teoría de las transiciones fue esencial para fundamentar los objetivos terapéuticos al apuntar los resultados de indicadores del proceso de cuidar: sentirse ligado, interactuar, estar situado y desarrollar confianza y *coping*. La maestría fue contemplada, pues, según la teoría, en la ocurrencia de la integración fluida entre el equipo de enfermeros en el apoyo al cuidado del binomio persona mayor y cuidadores, esenciales, ante situaciones de crisis como de la pandemia, el cuidado a la persona mayor se duplicó por ser el grupo más susceptible a las complicaciones de COVID-19. La comprobación de esto fue la reducción en el número de intervenciones después de las primeras semanas.

En cuanto a las limitaciones del estudio, puede decirse que en el auge de la pandemia el acceso a los datos de la persona mayor y sus cuidadores registrados en el SAD, se encontraban desactualizados lo que imposibilitó a la randomización del estudio. Estas limitaciones llevaron al equipo de investigación a auxiliar al equipo del SAD en la actualización de los registros de las personas mayores y de sus cuidadores adheridos al SAD, concomitantemente a la realización de la investigación. La escasez de datos sobre la COVID-19 también fue una limitación.

En relación con las implicaciones para el avance del conocimiento científico para el área de la salud y enfermería, observaron que la teleenfermería puede ser responsable de implicaciones positivas y negativas cuando es desarrollada por personas no expertas como un *call center*. Los cuidados de enfermería por medio de la teleenfermería pueden ser realizados en toda la red pública y privada de asistencia a la salud de la persona mayor, sus cuidadores y demás ciudadanos que necesiten las intervenciones de enfermería. La tecnología de apoyo a los enfermeros demanda competencias técnicas y científicas necesarias para la toma de decisiones clínicas acertadas. Los instrumentos de llamada basados en evidencias científicas son útiles para direccionar la planificación del cuidado a la persona mayor y sus cuidadores durante la pandemia de COVID-19. Las llamadas deben estar claramente enfocadas en la enfermedad y en el papel del enfermero en cuanto al cuidado longitudinal del binomio persona mayor y sus cuidadores asistidos por el Sistema de Atención Domiciliaria u otros contextos de salud.

La teleenfermería, a pesar de existir en el mundo, solo durante la pandemia de COVID-19 fue reglamentada en Brasil por el Sistema Cofen/Corens de forma emergencial para el apoyo de los enfermeros en la toma de decisiones clínicas. Ante esto, es fundamental que se desarrollen nuevas investigaciones sobre la efectividad de la teleenfermería como apoyo en la continuidad del cuidado integral a la salud de la persona mayor y sus cuidadores. El SAD, por ser un equipo de asistencia multidisciplinaria, para todo el equipo de salud, posibilitaría un seguimiento a la salud de sus registrados con diversas posibilidades de intervenciones positivas de apoyo a la continuidad del cuidado longitudinal^(33)-(34)^. 

## Conclusión

Los resultados demostraron la relevancia del uso de la cientificidad en cuanto a la combinación de la Teoría de Transición con la taxonomía de diagnóstico e intervenciones para la continuidad de la planificación de cuidados a la persona mayor y sus cuidadores asistidos por el SAD, a través de la teleenfermería, con una probabilidad de alta nueve veces mayor cuando se recibe el cuidado por teléfono. Los diagnósticos de enfermería evidenciados en este artículo como Riesgo de Tensión del Rol del Cuidador; Riesgo de Síndrome del Anciano Frágil y Riesgo de Contaminación, además de las intervenciones contenidas en el instrumento de llamada fueron eficientes y esenciales, pues permitieron atender al objetivo del estudio en el desarrollo de la teleenfermería como apoyo a la continuidad del cuidado presencial a remoto a la persona mayor y sus cuidadores enfocados en la prevención y promoción a la salud en el contexto pandémico. Así, el apoyo por medio de la teleenfermería también permitió al equipo multiprofesional del SAD un análisis preciso en cuanto a la alta con seguridad de los pacientes adheridos al servicio, derivándolos a la red de Atención Primaria a la Salud para atenciones de baja complejidad.
